# Interplay between the RBP SYNCRIP and RNA methylation in determining sEV miRNA-cargo and function in cell-to-cell communication

**DOI:** 10.1038/s41419-026-08991-9

**Published:** 2026-06-15

**Authors:** Luca Quattrocchi, Sabrina Garbo, Francesco Marocco, Maria Matilde Renne, Marco Loria, Marzia Pucci, Elisa Costanzo, Maria Felice Brizzi, Simona Fontana, Cecilia Battistelli, Marco Tripodi

**Affiliations:** 1 Department of Molecular Medicine, Department of Excellence 2023-2027, Sapienza University of Rome, Rome, Italy; 2 Department of Biomedicine, Neuroscience and Advanced Diagnostics (Bi.N.D) School of Medicine, University of Palermo, Palermo, Italy; 3 Department of Medical Sciences, University of Turin, Turin, Italy

**Keywords:** Epithelial-mesenchymal transition, Cancer models

## Abstract

Small extracellular vesicles (sEVs) are critical mediators of tumor microenvironment communication, largely through the selective transfer of microRNAs (miRNAs) that reprogram recipient cells. Active miRNA sorting into sEVs depends on RNA‑binding proteins (RBPs), sequence determinants, and RNA modifications. Here, a functional interplay between the RBP SYNCRIP and N6‑methyladenosine (m6A) RNA methylation controlling miRNA loading into hepatocellular carcinoma (HCC)‑derived sEVs has been disclosed. It is reported that (i) METTL3 (Methyltransferase-like-3)‑dependent m6A modification is required for efficient binding of SYNCRIP to specific miRNAs, thereby enabling their selective incorporation into sEVs; (ii) silencing of SYNCRIP markedly reshapes the sEV miRNA-cargo and impairs the ability of HCC‑derived sEVs to induce epithelial-to-mesenchymal transition (EMT) in non‑tumorigenic hepatocytes. Notably, (iii) depletion of METTL3 produces an even stronger effect, indicating that m6A methylation represents an upstream and essential determinant of SYNCRIP‑mediated miRNA export. Mechanistically, the data identify SYNCRIP as an m6A‑dependent miRNA reader, adding epitranscriptomic regulation to sequence‑based miRNA sorting into sEVs. Functionally, disruption of this interaction attenuates sEV‑driven EMT and pro‑tumorigenic signaling. Collectively, these findings uncover a novel regulatory axis governing sEV miRNA cargo selection and highlight the m6A–SYNCRIP interplay as a potential therapeutic target to interfere with sEV‑mediated tumor progression and metastasis.

## Introduction

Small extracellular vesicles (sEVs) represent a heterogeneous family of cell-derived non-replicating particles containing an internal informational cargo (e.g., proteins, DNA, coding and non-coding RNAs) influencing a wide range of physiological and pathological processes [1, 2].

In several types of cancer, active communication between tumor cells, the tumor microenvironment (TME), and neighboring cells is essential for tumor progression [3]. In this context, sEVs participate in the formation of pre-/pro-metastatic niche by remodeling the TME and promoting epithelial-to-mesenchymal transition (EMT), cell proliferation, metastasis formation, angiogenesis, and apoptosis through the transfer of oncogenic cargo in terms of proteins and RNAs [4, 5].

Concerning miRNAs, their export in the sEVs does not necessarily mirror their expression levels in the sEV-producing cells, thus indicating the existence of selective miRNA-loading mechanisms [6, 7].

Current evidence sheds light on specific RNA-binding proteins (RBPs) as well as on specific sequence determinants (consensus motifs) that contribute to miRNA sEV-loading [8]. In this regard, several components of the heterogeneous nuclear ribonucleoproteins (hnRNPs) family [9–14] have been reported to recognize specific motifs, mediating miRNAs delivery.

Specifically, the hnRNP SYNCRIP (synaptotagmin-binding cytoplasmic RNA-interacting protein) was disclosed as a sEV-miRNA interactor [12] recognizing the hEXO motif through its N-terminal unit for RNA recognition domain [13]. Notably, the inclusion of this motif in a cell-retained miRNA allowed its SYNCRIP-dependent sEV-loading [12].

Furthermore, SYNCRIP participates in the onset and development of several malignancies, promotes cell proliferation [15], and regulates the EMT/MET dynamics together with previously described transcription factors and ncRNAs [16–21]. Furthermore, in HCC invasive cells, its knockdown induces MET by regulating EMT-related miRNAs’ expression, repressing mesenchymal genes, inducing epithelial markers, and significantly impairing cell migration [22].

More recently, a further RBP (i.e., the pleiotropic PCBP2 (PolyC binding protein 2)) [23–25] has been recognized as a component of the multi-protein machinery regulating miRNA partition between sEVs and intracellular compartments. PCBP2 binds to the CELL motif enriched in intracellular-retained miRNAs and counteracts SYNCRIP-mediated sEV-loading of miRNAs sharing both the hEXO and the CELL motif [26].

In addition, recent research reported that the epitranscriptomic m6A modification also has an impact on ncRNAs. m6A influences the stability and expression levels of lncRNAs, impacting apoptosis, metabolism, and metastasis [27, 28] and is propaedeutic to lncRNA interaction with DNA/RBPs and thus to their function [29].

Moreover, m6A on mature miRNAs impacts their function and sEV-compartmentalization, by impairing AGO2 binding, and thereby miRNAs’ activity, and by enhancing miRNAs binding to the m6A-reader and sEV-loader hRNPA2B1, which facilitates their packaging into sEVs, ultimately influencing their fate [30].

With respect to m6A readers, they mediate different cellular processes, acting as oncogenes or tumor suppressors [31–37]. The majority of m6A readers display oncogenic activities in cancer; however, their functional roles are highly context-dependent, highlighting the complexity and therapeutic potential of targeting m6A regulatory pathways.

In this study, the role of SYNCRIP in reshaping the molecular cargo of sEVs in HCC, assessed by next-generation sequencing (NGS) and in mediating sEV-dependent induction of EMT, was clarified. Notably, several SYNCRIP-loaded sEV miRNAs are modified by m6A epitranscriptomic mark as evaluated by meRIP assay. Mechanistically, m6A is required for SYNCRIP binding as demonstrated by CLIP analysis, thereby revealing a previously unrecognized function of SYNCRIP not only as a miRNA sEV-loading factor but also as an m6A reader. Finally, the contribution of m6A methylation in determining HCC-derived sEV miRNA cargo in EMT induction was disclosed.

## Materials and methods

### Cell culture conditions

HepG2 cells were grown at 37 °C, in a humidified atmosphere with 5% CO_2_, in DMEM (Gibco-Life Technologies) supplemented with 10% fetal bovine serum (FBS) (Gibco-Life Technologies), L-Glutamine (2 mM), and Penicillin (100 U/ml)/Streptomycin (100 μg/ml) (Euroclone, UK). The colorectal cancer cell line SW480 (ATCC CCL 228) was maintained in RPMI 1640 medium (Euroclone, UK) supplemented with 10% FBS, 2 mM l-glutamine, 100 U/ml penicillin, and 100 µg/ml streptomycin.

THLE-2 cells were grown at 37 °C in humified atmosphere with 5% CO_2_, in Airway Epithelial Cell Basal Medium (ATCC, USA) with the Bronchial Epithelial Cell Growth Kit (ATCC, USA) supplemented with 70 ng/ml phosphoethanolamine, 5 ng/ml epidermal growth factor, 10% FBS, 100 U/ml penicillin, and 100 µg/ml streptomycin. Cells were cultured in pre-coated flasks, containing 0.01 mg/ml fibronectin (Sigma-Aldrich, USA), 0.03 mg/ml bovine collagen type I (Advanced Biomatrix, USA), and 0.01 mg/ml bovine serum albumin (Sigma-Aldrich, USA).

### Gene silencing

Stable SYNCRIP knockdown was achieved through infection with SMARTvector Lentiviral shRNA (Dharmacon Horizon, USA). Viral supernatants were collected 48 h after transfection of HEK293T packaging cells, filtered (0.45 μm), and added to HepG2 cells. At 24 h post-infection, selection was performed with 2 μg/mL puromycin (Sigma-Aldrich, USA) for 1 week. Inducible shRNAs’ expression was obtained by doxycycline treatment (2 μg/mL) for 72 h.

The sequences of shRNA oligos used for silencing and relative controls are reported in Table 1.

**Table 1:** Sequences of shRNA oligos used for protein silencing and relative controls.

Name	Sequence
shCTR#1	As reported in [12]
shSYNCRIP	TTAGGAATAGAGCCCACAA
shCTR#2	As reported in [30]
shMETTL3	As reported in [30]

### SDS-PAGE and western blotting

Cells were lysed in Triton 1X Buffer, and the proteins were analyzed as in [21]. The following primary antibodies were used for immunoblotting: a-SYNCRIP (MAB11004 Merck Millipore, Germany), a-METTL3 (ab195352 Abcam, UK), a-LAMP1 (ab24170 Abcam, UK), a-TSG101 (sc7964 Santa Cruz Biotechnology, USA), a-CD63 (sc5275 Santa Cruz Biotechnology, USA), a-Flotillin (sc74566 Santa Cruz Biotechnology, USA), a-CD81 (ab109201 Abcam, UK), a-CALNEXIN (NB100-1965 Novus Biologicals, USA) and a-GAPDH (MAB-374 Merck Millipore, Germany). The immune complexes were detected with horseradish peroxidase-conjugated species-specific secondary antiserum (α-Rabbit 172-1019 and α-Mouse 170-6516; Bio-Rad Laboratories Inc., USA), then by enhanced chemiluminescence reaction (Bio-Rad Laboratories Inc., USA). Densitometric analysis of protein expression was performed by using the Fiji-ImageJ image processing package.

### sEV purification

sEVs were prepared according to ISEV recommendations [38, 39]. Conditioned media were collected after 72 h of culture in a complete medium containing sEVs-depleted FBS. Cell-conditioned media were centrifuged at 2000 × *g* for 20 min at 4 °C to remove dead cells and then at 20,000× *g* for 30 min at 4 °C to remove microvesicles. Cleared supernatants were passed through 0.22 μm filter membranes, ultracentrifuged in a SW32 Ti rotor (Beckman Coulter, USA) at 100,000 × *g* for 70 min at 4 °C, washed with PBS at 100,000 × *g* for 70 min at 4 °C, and finally resuspended in PBS. The sEVs resuspension was analyzed by Tunable Resistive Pulse Sensing technology using EXOID-V1-SC (Izon Science, New Zealand) for size and concentration characterization and by Western blot assay.

### RNA extraction, RT-PCR, and real-time qPCR

miRNAs were extracted by miRNeasy Mini Kit and RNeasy MinElute Cleanup Kit (QIAGEN, Germany), and reverse transcribed with microScript microRNA c-DNA Synthesis Kit (Norgen Biotek Canada). Quantitative polymerase chain reaction (RT-qPCR) analyses were performed according to MIQE guidelines [40, 41]. cDNAs were amplified by RT-qPCR reaction using GoTaq qPCR Master Mix (Promega Corporation, USA). Relative amounts, obtained with the 2^(−ΔCt)^ method, were normalized with respect to the cel-miR-39 Spike-In (Norgen Biotek, Canada), previously added to miRNA samples.

Total RNA was extracted by ReliaPrepTM RNA Tissue Miniprep System (Promega Corporation, USA) and reverse transcribed with iScriptTM c-DNA Synthesis Kit (Bio-Rad Laboratories Inc., USA). cDNAs were amplified by RT-qPCR reaction using GoTaq qPCR Master Mix (Promega Corporation, USA). Relative amounts, obtained with the 2^(−ΔCt)^ method, were normalized with respect to the housekeeping gene L32.

Oligonucleotide sequences for miRNAs and mRNA expression analysis are reported in Table 2 and Table 3, respectively.

**Table 2:** Oligonucleotide sequences for miRNA expression analysis.

Name	Sequence
cel-miR-39	TCACCGGGTGTAAATCAGCTTG
hsa-miR-26a-5p	TTCAAGTAATCCAGGATAGGCT
hsa-miR-103a-3p	AGCAGCATTGTACAGGGCTATGA
hsa-miR-106b-5p	TAAAGTGCTGACAGTGCAGAT
hsa-miR-125b-5p	TCCCTGAGACCCTAACTTGTGA
hsa-miR-130a-3p	CAGTGCAATGTTAAAAGGGCAT
hsa-miR-130b-3p	CAGTGCAATGATGAAAGGGCAT
hsa-miR-143-3p	TGAGATGAAGCACTGTAGCTC
hsa-miR-194-5p	TGTAACAGCAACTCCATGTGGA
hsa-miR-342-3p	TCTCACACAGAAATCGCACCCGT
hsa-miR-369-3p	AATAATACATGGTTGATCTTT
hsa-miR-451a	AAACCGTTACCATTACTGAGTT
hsa-miR-483-3p	TCACTCCTCTCCTCCCGTCTT
hsa-miR-6529-5p	GAGAGATCAGAGGCGCAGAGTG
hsa-miR-6767-5p	TCGCAGACAGGGACACATGGAGA
hsa-miR-1180-3p	TTTCCGGCTCGCGTGGGTGTGT

**Table 3:** Oligonucleotide sequences for mRNA expression analysis.

Name	Sequence
SYNCRIP FW	CAAAAAGCCTATTGTTGGCA
SYNCRIP REV	AGCAGCTCAGGAGGCTGTTA
METTL3 FW	CTTGCATGGATTCTGAGGCC
METTL3 REV	GTCAGCCATCACAACTGCAA
L32 FW	GGAGCGACTGCTACGGAAG
L32 REV	GATACTGTCCAAAAGGCTGGAA
CK8 FW	ACCCAGGAGAAGGAGCAGAT
CK8 REV	CTTGAAGTCCTCCACCAGCC
E-CADHERIN FW	TACGCCTGGGACTCCACCTA
E-CADHERIN REV	CCAGAAACGGAGGCCTGAT
OCCLUDIN FW	AAGGTCAAAGAGAACAGAGCAAGA
OCCLUDIN REV	TATTCCCTGATCCAGTCCTCCTC
SNAIL FW	CACTATGCCGCGCTCTTTC
SNAIL REV	GCTGGAAGGTAAACTCTGGATTAGA
VIMENTIN FW	GCTAACCAACGACAAAGCCC
VIMENTIN REV	GATTGCAGGGTGTTTTCGGC
α-SMA FW	CAGCCAAGCACTGTCAGG
α-SMA REV	CCAGAGCCATTGTCACACAC
CK18 FW	GAGGGCTCAGATCTTCGCAA
CK18 REV	TGGCAATCTGGGCTTGTAGG

### Small RNA-seq

miRNA samples were sequenced at Procomcure Biotech GmbH. The sequencing experiment included three independent biological replicates for the control (shCTR) condition and four biological replicates for the knockdown (shSYNCRIP) condition. Sequencing libraries were prepared using the NEXTFLEX Small RNA-Seq Kit v4 (PerkinElmer, USA). The sequencing reaction was performed on an Illumina NovaSeq 6000 instrument in 2 × 40 bp paired-end configuration, with a throughput of ~40 million read pairs per sample. FastqToolkit version 2.2.5 was used to remove adapter sequences from the 3′ end, whereas Cutadapt was used to filter out reads whose length and average quality after trimming were <16 and <30, respectively. Only forward reads were kept for downstream analyses. The MiRDeep2 software version 1.3.1 was used to align and quantify the reads against a reference composed of precursor and mature miRNA sequences downloaded from the miRBase v22.1 database. Raw mapped counts were then processed for differential abundance analysis using the limma-voom pipeline within the edgeR framework. Counts were filtered, normalized using the trimmed mean of *M* values (TMM) method, and precision weights were estimated using the voom function, followed by linear modeling and empirical Bayes moderation. Differentially abundant miRNAs were defined based on a nominal *p* value threshold of <0.1 and a fold-change cutoff of ∣FC∣ > 0.5. Adjusted *p* values calculated via the Benjamini–Hochberg false discovery rate (FDR) procedure were also reported in the Supplementary material.

### Target prediction and functional analysis

The prediction of the genes targeted by the miRNAs and the subsequent functional enrichment analysis were performed using a pipeline developed in-house. In detail, the prediction of miRNA target genes was performed using the multiMiR package (version 1.24.0) in R.

Experimentally validated and predicted miRNA-target interactions were retrieved from the multiMiR database. To obtain a target list, all validated interactions were combined with only those predicted (≥2 databases) among the ones included in multiMiR.

Gene Ontology (GO) and Kyoto Encyclopedia of Genes and Genomes (KEGG) enrichment analyses were performed using the clusterProfiler package in R, with gene annotation information obtained from the org.Hs.eg.db database.

For both analyses, *p* values were adjusted for multiple testing using the Benjamini–Hochberg FDR correction. Terms or pathways with an adjusted *p* value (FDR) < 0.05 were considered statistically significant.

Enriched GO terms and KEGG pathways were filtered to retain only those functionally associated with EMT.

Enrichment results were visualized as dot plots generated with ggplot2, highlighting EMT-related GO and KEGG terms.

### Motif search analysis

The motif search analysis was performed to identify the presence of the hEXO motif, according to the IUPAC nucleotide code, as DDDVWS (D = not C; V = not U; W = U/A; S = G/C), within the sequences of the downregulated miRNAs (shSYNCRIP sEVs vs shCTR sEVs) obtained from the small RNA-seq dataset.

The analysis was carried out using Python (version 3.12.7) and regular expressions (regex) to scan the mature miRNA sequences for the presence of the motif of interest.

Since the hEXO motif is reported to occur outside the seed region, the first seven nucleotides at the 5′ end of each miRNA sequence were excluded from the search.

### Treatment of THLE-2 cells with sEVs

THLE-2 cells were treated with different sEV samples. After reaching subconfluence, THLE-2 cells were treated for 24 h with approximately 1.5 × 10^10^ sEVs produced by SW480 (used as a positive control of EV-mediated EMT induction in THLE-2 as in [5] and 0.6 × 10^10^ particles/ml of sEVs derived from HepG2, both shCTR and shSYNCRIP or shCTR and shMETTL3).

### Confocal fluorescence microscopy

THLE-2 were fixed with 4% PFA, permeabilized for 5 min with 0.1% Triton X-100; after blocking for 1 h with BSA 3%, cells were incubated for 1 h with anti-Vimentin (Cell Signaling Technology, USA) and anti-CK8/18 (Cell Signaling Technology, USA), then cells were incubated with DyLight 594 secondary antibody (Thermo Fisher Scientific, USA). Nuclei were stained with Hoechst (Molecular Probes, Life Technologies, USA), and F-actin was stained with Actin Green (Thermo Fisher, USA). The samples were analyzed by confocal fluorescence microscopy (Nikon A1 confocal microscope, Japan).

### Methylated RNA immunoprecipitation (meRIP)

meRIP was performed in accordance with [30]. Antibody recognizing m6A (202 003 Synaptic System, Germany) or Normal Rabbit IgG (12–370; Millipore, Germany) was added to the miRNA sample diluted in IP buffer (10 mM Tris [pH 7.5], 150 mM NaCl, 0.5% Nonidet P-40) and then incubated with protein A magnetic beads. miRNAs were eluted with elution buffer (5 mM Tris-HCl, pH 7.5, 1 mM EDTA, 0.05% SDS), extracted using the phenol-chloroform method, and then analyzed with qRT-PCR. The list of primers is reported in Table 2.

### UV cross-linking RNA immunoprecipitation (CLIP)

UV cross-linking CLIP was performed as reported in [21]. Immunoprecipitated miRNAs were reverse transcribed and analyzed by RT-qPCR amplification. Primary antibodies: a-SYNCRIP (MAB11004 – Merck Millipore, Germany) and, as a negative control, Normal Mouse IgG (12-371 Merck Millipore, Germany). The list of primers used in qPCR analysis is reported in Table 2.

### Biotin-miRNA pull-down

Biotin miRNA pull-down experiments were performed on cytoplasmic extracts. Briefly, cells were lysed in hypotonic buffer (10 mM Tris-Cl [pH 7.5], 20 mM KCl, 1.5 mM MgCl2, 5 mM DTT, 0.5 mM EGTA, 5% glycerol, 0.5% NP40, and 40 U/mL RNAsin [Promega]) supplemented with protease inhibitors (Roche Applied Science). Lysates were incubated on a rotating platform for 30 min at 4 °C and then centrifuged at 13,000 rpm for 30 min at 4 °C. Protein concentration was determined with Protein Assay Dye Reagent (Bio-Rad) based on the Bradford assay. Samples (1 mg of proteins) were incubated for 1 h at 4 °C with 10 nmol synthetic single-strand miRNA oligonucleotides containing a biotin modification attached to the 5’ and via a spacer arm (IDT, Integrated DNA Technology). Dynabeads M-280 Streptavidin (50 μL/sample, Invitrogen), previously blocked with 1 mg/mL yeast tRNA (Roche Applied Science), were added to the reaction mixture for 90 min at 4 °C, and then the beads were washed three times with cold lysis buffer and once with PBS. Elution was performed at room temperature for 5 min in Laemmli Buffer (containing 2-β mercaptoethanol and SDS). Detection of miRNA/RBPs interaction was evaluated by WB on 10% of the input sample and 50% of the pulled-down samples.

Biotin-miRNAs sequences:

-has-miR-6767-5p (/5BiosG/UCGCAGACAGGGACACAUGGAGA3′)

-has-miR-6767-5p m6A (/5BiosG/UCGCAGM6ACAGGGM6ACACAUGGAGA3′)

-has-miR-103a-3p (/5BiosG/AGCAGCAUUGUACAGGGCUAUGA3′)

- has-miR-103a-3p (/5BiosG/AGCAGCAUUGUM6ACAGGGCUAUGA)

### Scratch assay

THLE-2 cells were maintained in culture medium, then shifted to serum-depleted culture medium to inhibit cell proliferation. A scratch wound was created on the cell layer using a micropipette tip. Micrographs were taken at 0 and 48 h after the scratch. Cell-devoid areas at time 0 and 48 h after the scratch were quantified through the Fiji-ImageJ image processing package.

### Statistical analysis

Statistical analysis was performed using paired Student’s *t*-tests. To account for multiple comparisons, *p* values were adjusted using the Benjamini–Hochberg FDR procedure. Controls included solely for assay validation (e.g., negative or methylation-independent controls) were not part of the primary inferential hypotheses and were therefore excluded from multiple testing correction. All statistical analyses were conducted using GraphPad Prism Version 9 (GraphPad Software). Data are presented as mean ± S.E.M., and adjusted *p* values < 0.05 were considered statistically significant. Significance levels are indicated as follows: ∗*p* < 0.05; ∗∗*p* < 0.01; ∗∗∗*p* < 0.001.

## Results

### HCC cell-derived sEVs-miRNA cargo is deeply impacted by SYNCRIP and is predicted to target EMT-related genes

**Fig. 1:**
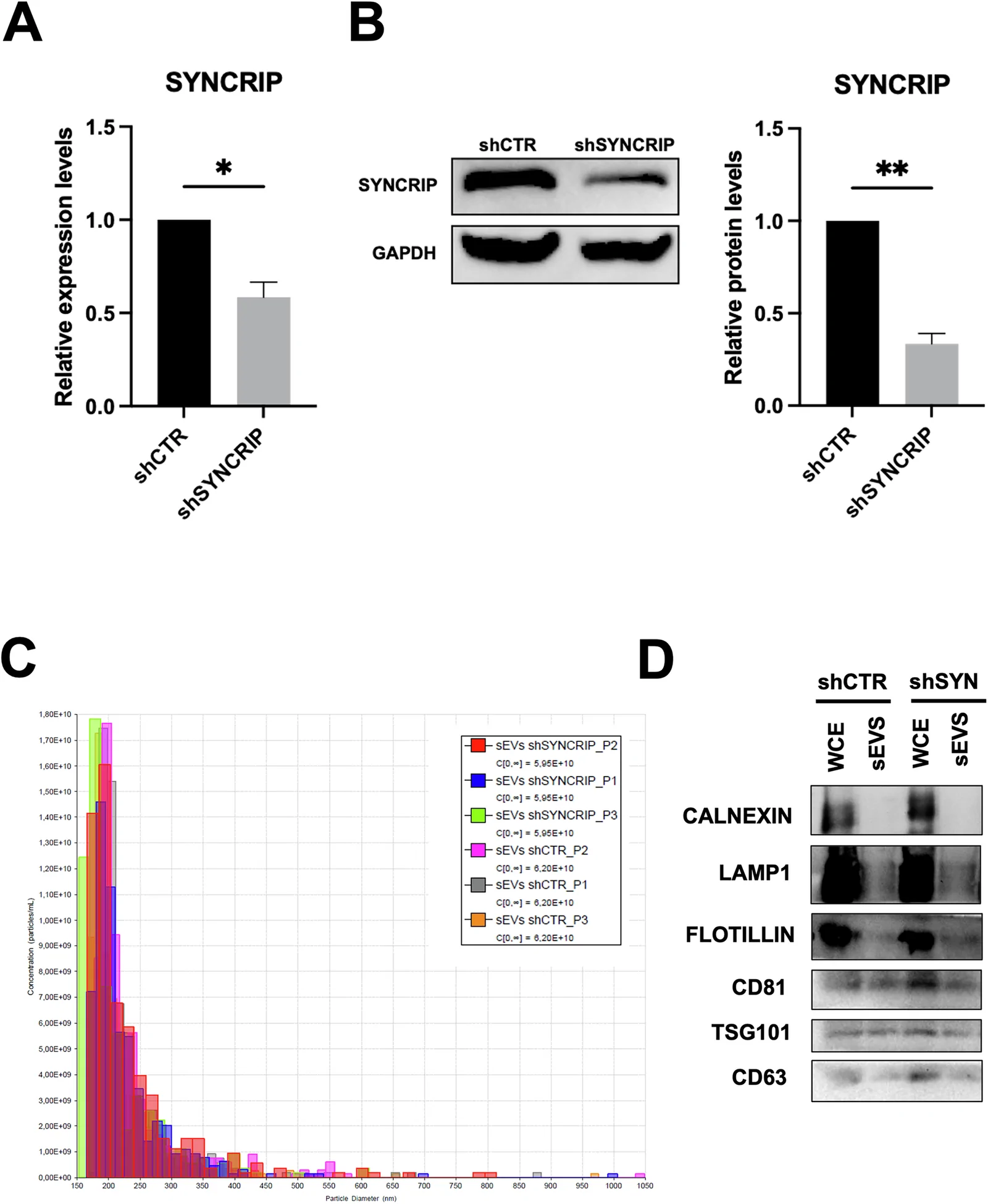
SYNCRIP silencing in HepG2 cells and sEV characterization. **A** Expression levels of SYNCRIP in shCTR and shSYNCRIP HepG2 HCC cells assessed by RT-qPCR. Data are shown as the mean ± S.E.M. of three independent experiments. Data were considered statistically significant with *p* < 0.05 (*p < 0.05). **B** (Left panel) Western blot analysis for SYNCRIP on protein extracts from HepG2 cells silenced for SYNCRIP (shSYNCRIP) and relative control (shCTR). GAPDH has been used as a loading control. The figure is representative of three independent experiments. (Right panel) Densitometric analysis of Western blot signals. Data are shown as the mean ± S.E.M. of three independent experiments. Data were considered statistically significant with *p* < 0.05 (**p < 0.01). **C** Particle diameter (nm) and concentration (particles/ml) of sEVs evaluated by Exoid (IZON). Histograms report the concentration (particles/ml) by size (nm) for each of the two analyzed conditions, as in (**A**). For each condition, three different measurements were performed (P1, P2, and P3). **D** Western blot analysis for sEV-specific (LAMP1- Flotillin- CD81- TSG101- CD63) and intracellular (CALNEXIN) markers on protein extracts from HepG2 cells-derived sEVs (sEVs) and HepG2 cells (both shCTR and shSYNCRIP). (WCE whole cell extract).

To investigate the role and involvement of SYNCRIP in the sEV-mediated promotion and development of the TME, HCC cells (HepG2) silenced for SYNCRIP were established (Fig. 1A, B) by expressing a short hairpin RNA targeting SYNCRIP or a scrambled sequence as a negative control (Table 1).

Both HepG2 shCTR and shSYNCRIP cells were cultured to collect sEVs, analyzed by particle size distribution, concentration, and western blot analysis for recognized cellular and EV markers (Fig. 1C, D).

**Fig. 2:**
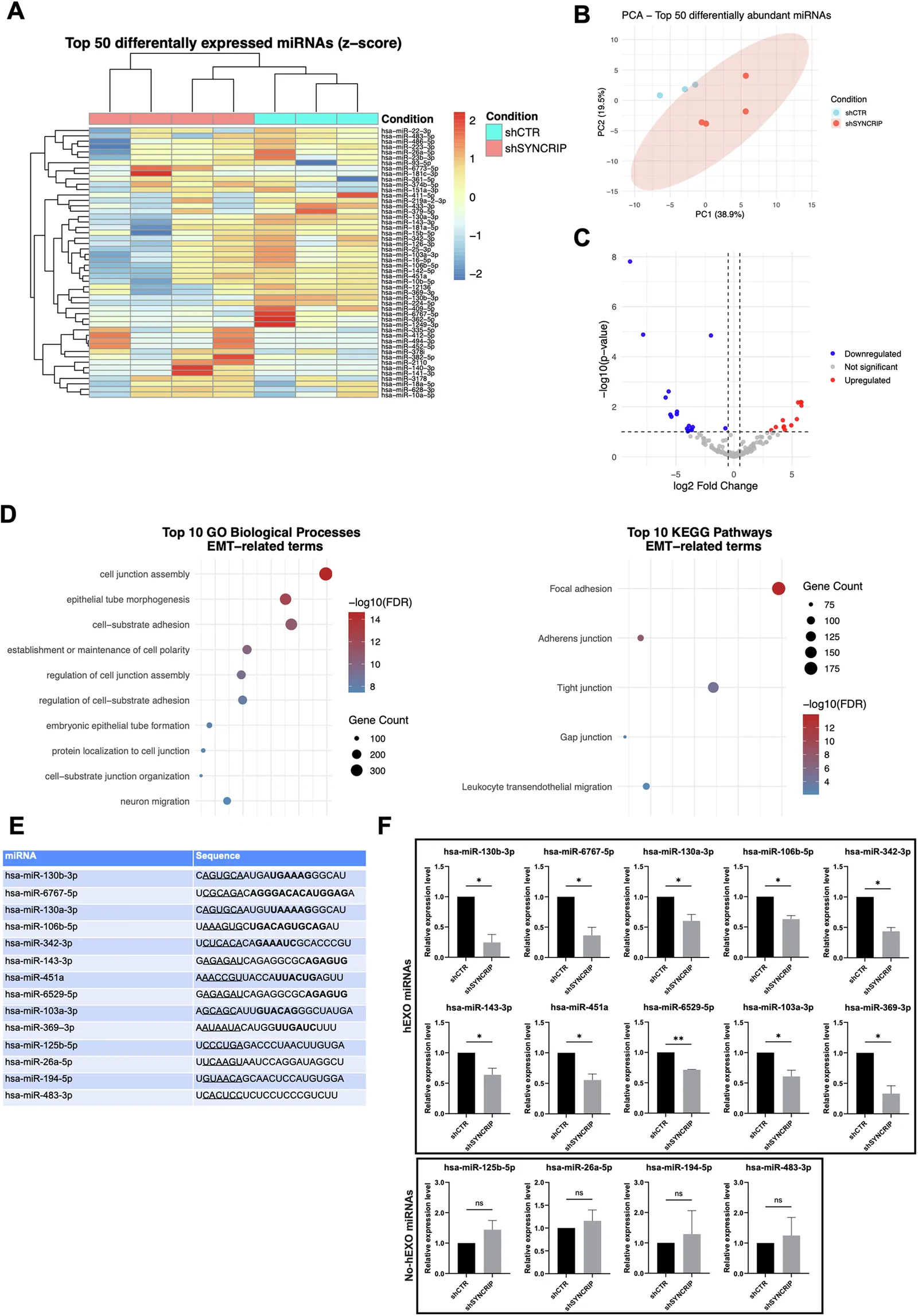
HCC cell-derived sEVs-miRNA cargo is deeply impacted by SYNCRIP and is predicted to target EMT-related genes. **A** Heatmap of the top 50 differentially abundant microRNAs (shCTR vs shSYNCRIP). Values represent RNA-seq-derived miRNA expression, normalized and scaled per miRNA as *z*-scores. Colors indicate relative expression levels (blue = low; white = average; red = high). Hierarchical clustering was applied to both miRNAs and samples. **B** Principal component analysis (PCA) of the top 50 differentially abundant microRNAs (shCTR and shSYNCRIP). **C** Volcano plot of differentially abundant microRNAs. Cutoffs for differential abundance were set at a nominal *p* value < 0.1 and a fold-change ∣FC∣ > 0.5. **D** SYNCRIP-dependent sEV-miRNAs target prediction indicating enrichment in EMT-related terms. (Left) Gene Ontology and (Right) KEGG enrichment analysis on validated and predicted targets. The *X*-axis represents the gene ratio; the *Y*-axis represents the enriched terms. Colors (blue = lower, red = higher statistical significance) indicate the statistical significance after multiple test correction, while circle size represents the number of genes associated with each term. **E** Motif search analysis on the differentially exported miRNAs. SYNCRIP binding site is highlighted in bold, seed sequence is underlined. **F** Expression levels of differentially abundant miRNAs assessed by RT-qPCR in sEVs derived from control (shCTR) and silenced (shSYNCRIP) HCC cells. Data are shown as the mean ± S.E.M. of three independent experiments and are considered statistically significant with *p* < 0.05 (*p < 0.05; **p < 0.01; ***p < 0.001; ns not statistically significant).

To characterize the pool of miRNAs selectively exported into sEVs in a SYNCRIP-dependent manner, miRNAs were analyzed by NGS. Data revealed a group of candidates whose abundance in sEVs was consistently reduced upon SYNCRIP silencing (Fig. 2A–C). Target prediction by MultimiR identified several key genes previously reported as involved in the regulation of EMT [21]. Furthermore, functional analysis by both GO and KEGG highlighted the enrichment of several processes associated with EMT. Additionally, the enrichment results for EMT-related keywords revealed processes involved in epithelial phenotype maintenance (e.g., cell junction assembly, cell-substrate adhesion, maintenance of cell polarity) (Fig. 2D).

Thus, the emerging trend strongly suggests that SYNCRIP acts as a determinant of selective miRNA loading into sEVs also in HCC cells and that several of these SYNCRIP-dependent sEV-loaded miRNAs are involved in EMT induction.

Coherent with previous findings [12], a downstream motif analysis on the candidate miRNAs revealed an enrichment in the hEXO motif, shared among SYNCRIP-exported miRNAs (Fig. 2E).

To validate the wide range of data, a subset of miRNAs embedding the hEXO motif was selected to be analyzed by RT-qPCR. As negative controls, miRNAs lacking a hEXO motif were used, and their export into sEVs did not change upon SYNCRIP silencing. The RT-qPCR analysis confirmed the sEV small RNA-seq data, demonstrating that several miRNAs embedding the hEXO motif are significantly less exported in sEVs released from SYNCRIP-silenced HCC cells compared to controls (Fig. 2F).

Taken together, these results support the conclusion that SYNCRIP exerts a key role in controlling the molecular composition of the sEV-miRNA cargo in HCC cells and suggest the need to investigate their functional properties.

### sEV-mediated EMT requires SYNCRIP activity

Based on the SYNCRIP intracellular role in HCC as an EMT inducer [22], on its function in sEVs cargo (miRNAs) loading (Fig. 2A, F), and on the miRNA target predictions (Fig. 2D), its influence on EMT onset was hypothesized to be also due to its miRNAs sEV-loading function. Thus, SYNCRIP sEV-mediated functional activity was analyzed by evaluating the impact on miRNA-targets’ expression in sEV-receiving cells.

For this purpose, human THLE-2 non-tumorigenic hepatocytes were treated with sEVs derived from control or from SYNCRIP-silenced cells to evaluate changes in EMT-marker expression following the treatment.

**Fig. 3:**
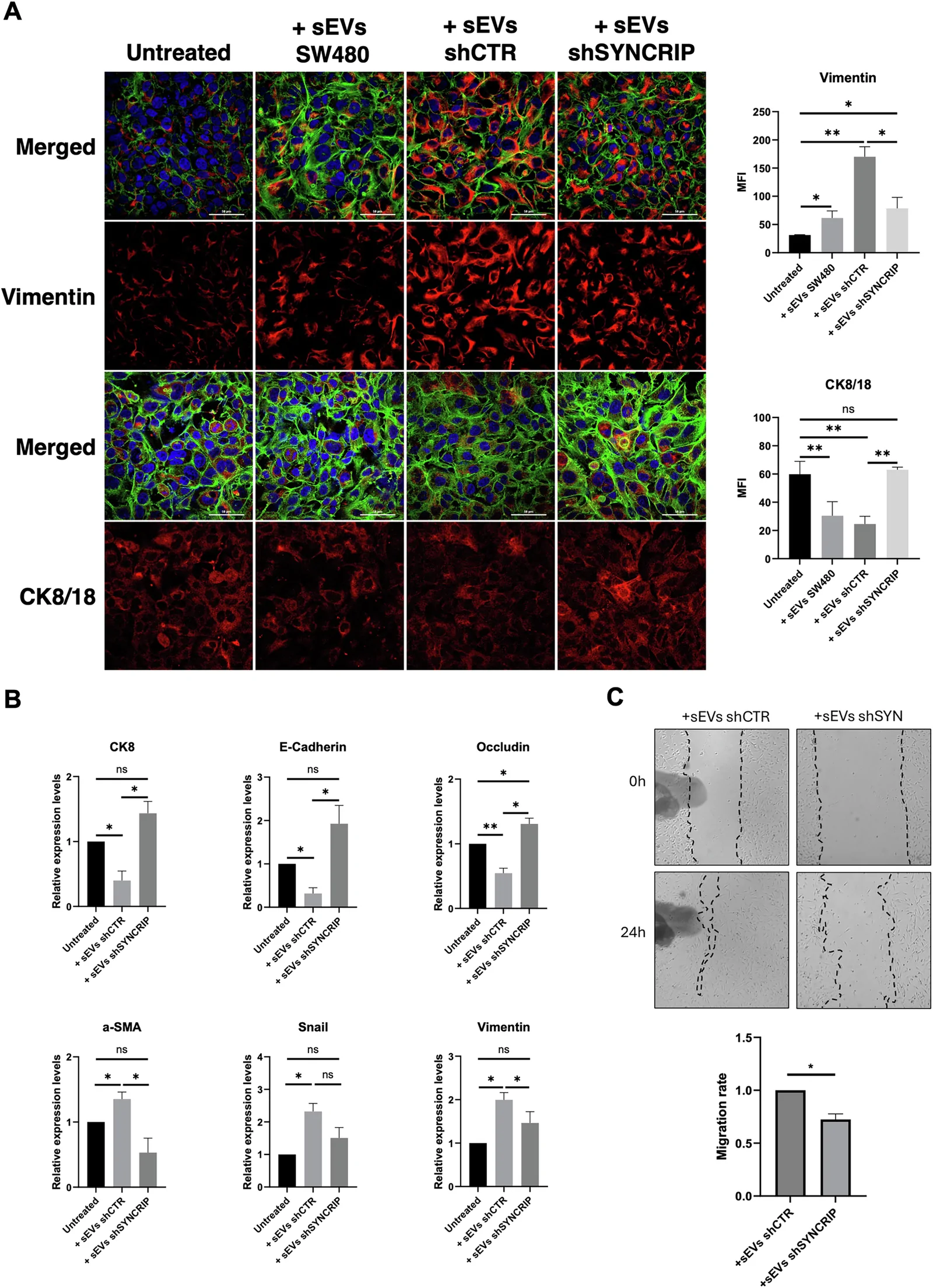
sEV-mediated EMT requires SYNCRIP activity. **A** (Left) Immunofluorescence assay on THLE-2 hepatocytes treated with the indicated sEVs. sEVs from SW480 colorectal cancer cells are used as a positive control of sEV-mediated EMT induction, as in [5]. Nuclei (blue), actin (green), Vimentin, and CK8/18 (red). Results are representative of four independent experiments. Scale bar: 50 μm. (Right) Quantitative analysis of immunofluorescence signals (MFI mean fluorescence intensity). Data are shown as the mean ± S.E.M. of four independent experiments. **B** RT-qPCR on EMT markers on RNA samples derived from THLE-2 hepatocytes treated with the indicated sEVs. (Top) epithelial markers; (Bottom) mesenchymal markers. Data are shown as the mean ± S.E.M. of three independent experiments. **C** (Top) Scratch assay at the indicated time in human non-tumorigenic hepatocytes treated with sEVs derived from shCTR or shSYNCRIP HepG2 cells. The figure is representative of three independent experiments. For each experimental replicate, the same randomly chosen field has been monitored over time. (Bottom) Quantification of migration, data are shown as the mean ± S.E.M of three independent experiments. **A**–**C** Data are considered statistically significant with *p* < 0.05 (*p < 0.05; **p < 0.01; ns not statistically significant).

Following the bioinformatic prediction, the expression of EMT markers was examined both by immunofluorescence and RT-qPCR. Hepatocytes treated with control sEVs undergo EMT (i.e., reduction of the expression of the epithelial marker CK8/18 and induction of the expression of the mesenchymal marker vimentin). In contrast, hepatocytes exposed to sEVs produced by SYNCRIP-silenced cells displayed a markedly attenuated EMT induction: epithelial markers’ expression was preserved, while the induction of the mesenchymal markers was impaired (Fig. 3A).

Coherently, RT-qPCR highlighted that control sEVs induce EMT by reducing the expression of Cytokeratin 8, E-cadherin, and Occludin and by increasing the expression of Vimentin, Snail, and αSMA; conversely, this effect was not observed upon treatment with sEVs produced by shSYNCRIP HCC cells (Fig. 3B). miRNA (103a-3p, 106b-5p, 130b-3p) mimics’ transfection confirmed their direct capacity to target the analyzed EMT-related markers (Supplementary Fig. 1). These findings confirm that distinct miRNAs sorted in sEVs by SYNCRIP are specific regulators of EMT-related genes.

We further investigated the functional consequences of sEV-uptake on the migratory capacity of THLE-2 cells. Consistent with the observations on gene expression regulation, treatment with sEVs derived from both shCTR and shSYNCRIP HCC cells significantly promoted THLE-2 cell migration compared to untreated controls. Notably, the pro-migratory effect was markedly attenuated in cells treated with sEVs from SYNCRIP-depleted HCC cells. These results suggest that while HCC-derived sEVs generally enhance the mobility of non-tumorigenic hepatocytes, the knockdown of SYNCRIP in EV-producing cells alters the sEV cargo that diminishes the migration induction (Fig. 3C).

Thus, SYNCRIP knockdown in sEV-producing cells results in EMT impairment in sEV-recipient hepatocytes compared to the control counterpart.

Altogether, SYNCRIP promotes intercellular communication by shaping sEV miRNA cargo, which drives non-tumorigenic epithelial hepatocytes toward an EMT-like state; conversely, its silencing diminishes this effect, thereby limiting the acquisition of mesenchymal traits in recipient cells.

Overall, these data demonstrate SYNCRIP’s contribution to the modulation of sEV-miRNA cargo, and in turn, to the sEV-dependent pro-EMT induction.

### Several SYNCRIP sEV-loaded miRNAs are m6A-modified, and their targets are enriched in EMT-related terms

**Fig. 4:**
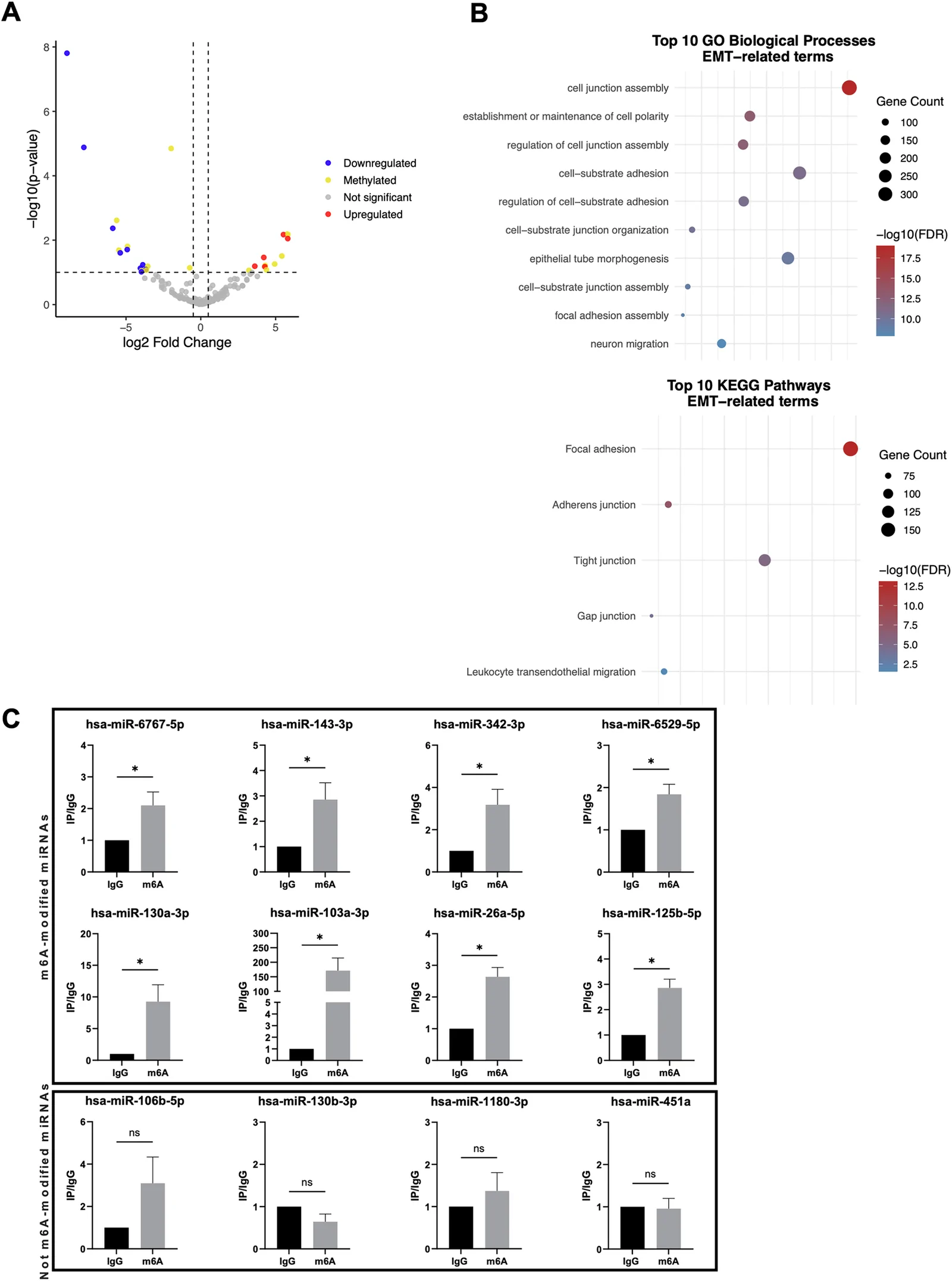
Several SYNCRIP sEV-loaded miRNAs are m6A-modified, and their targets are enriched in EMT-related terms. **A** Volcano plot highlighting miRNAs target of SYNCRIP and METTL3 (m6A methylated). **B** (Top) Gene Ontology and (Bottom) KEGG enrichment analysis on validated and predicted targets of the methylated miRNAs targeted by SYNCRIP. The *X*-axis represents the gene ratio; the *Y*-axis represents the enriched terms. Colors and circle size significance as in Fig. 2D. **C** meRIP-seq validation: meRIP-qPCR analysis on miRNAs from HepG2 cells. Data are shown as the mean ± S.E.M. of four independent experiments and are represented as IP/IgG. Data were considered statistically significant with *p* < 0.05 (*p < 0.05; **p < 0.01; ns not statistically significant).

Small RNA sequencing data from sEV samples (produced by both shCTR and shSYNCRIP HepG2 cells) integrated with available m6A immunoprecipitation sequencing (meRIP-seq) data [30] indicated that several of the SYNCRIP-exported miRNAs identified and validated by RT-qPCR undergo m6A modification (Fig. 4A).

Furthermore, a bioinformatic prediction of the genes targeted by methylated sEV-miRNAs identified several genes overlapping those previously found as targets of the SYNCRIP-regulated ones. This observation suggested the existence of downstream effectors under the coordinated control of both SYNCRIP and the m6A methylation machinery. Consistently, GO and KEGG enrichment analyses performed on these target genes revealed a significant enrichment of multiple pathways and biological processes associated with EMT (Fig. 4B).

meRIP-seq data on several miRNAs were confirmed by a meRIP followed by RT-qPCR (Fig. 4C).

This evidence suggests that m6A methylation on miRNAs is a key determinant of SYNCRIP-mediated sEV-export.

### SYNCRIP functions as a miRNA m6A reader

**Fig. 5:**
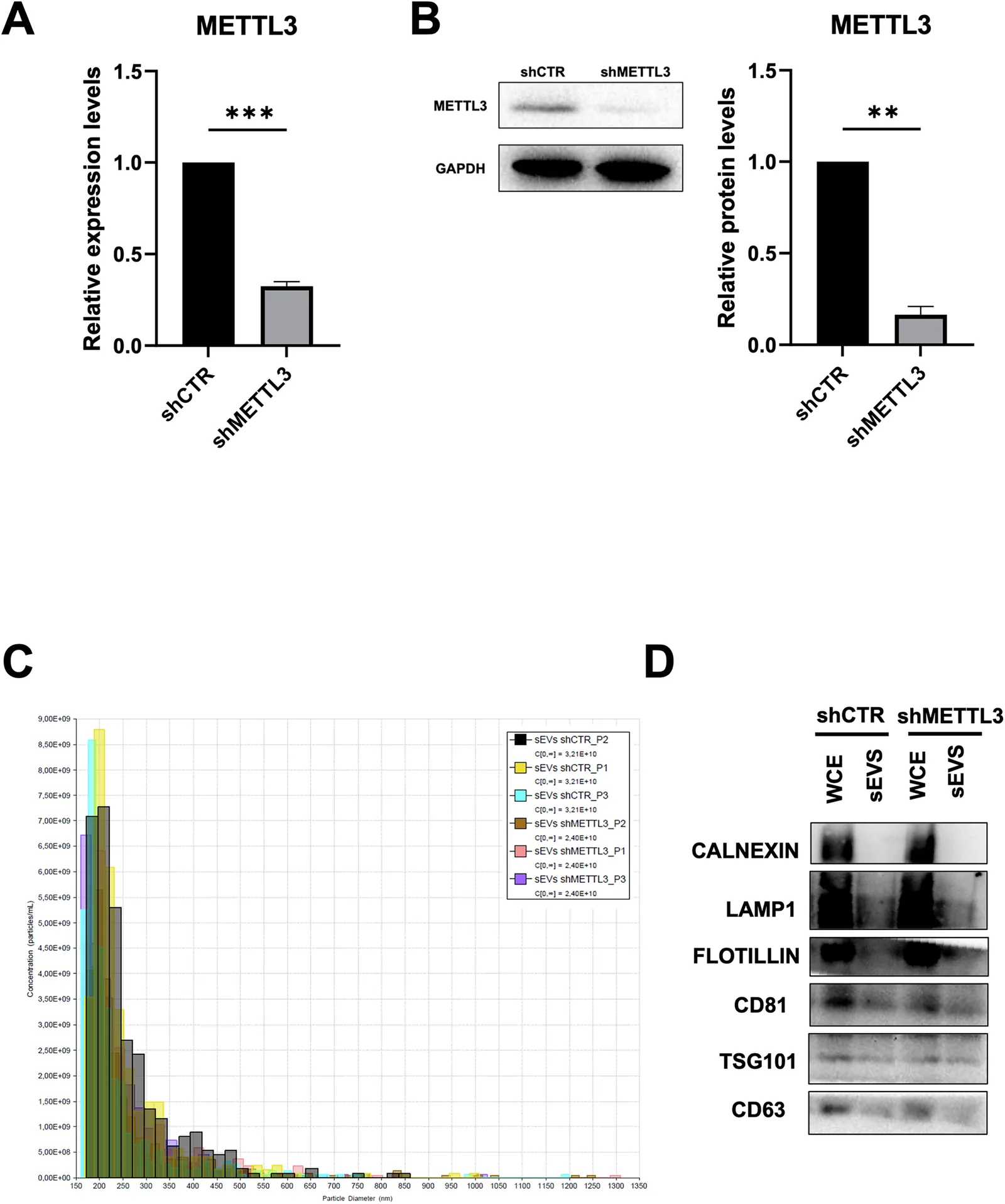
METTL3 silencing in HepG2 cells and sEV characterization. **A** Expression levels of METTL3 in shCTR and shMETTL3 HepG2 HCC cells assessed by RT-qPCR. Data are shown as the mean ± S.E.M. of three independent experiments. Data were considered statistically significant with *p* < 0.05 (*p < 0.05). **B** (Left) Western blot analysis for METTL3 on protein extracts from HepG2 cells silenced for METTL3 (shMETTL3) and relative control (shCTR). GAPDH has been used as a loading control. The figure is representative of three independent experiments. (Right) Densitometric analysis of Western blot signals. Data are shown as the mean ± S.E.M. of three independent experiments. Data were considered statistically significant with *p* < 0.05 (**p < 0.01). **C** Particle diameter (nm) and concentration (particles/ml) of sEVs evaluated by Exoid (IZON). Histograms report the concentration (particles/ml) by size (nm) for each of the two analyzed conditions. For each condition, three different measurements were performed (P1, P2, and P3). **D** Western blot analysis for sEV-specific (LAMP1- Flotillin- CD81- TSG101- CD63) and intracellular (CALNEXIN) markers on protein extracts from HepG2 cells-derived sEVs (sEVs) and HepG2 cells (both shCTR and shMETTL3). (WCE, whole cell extract).

**Fig. 6:**
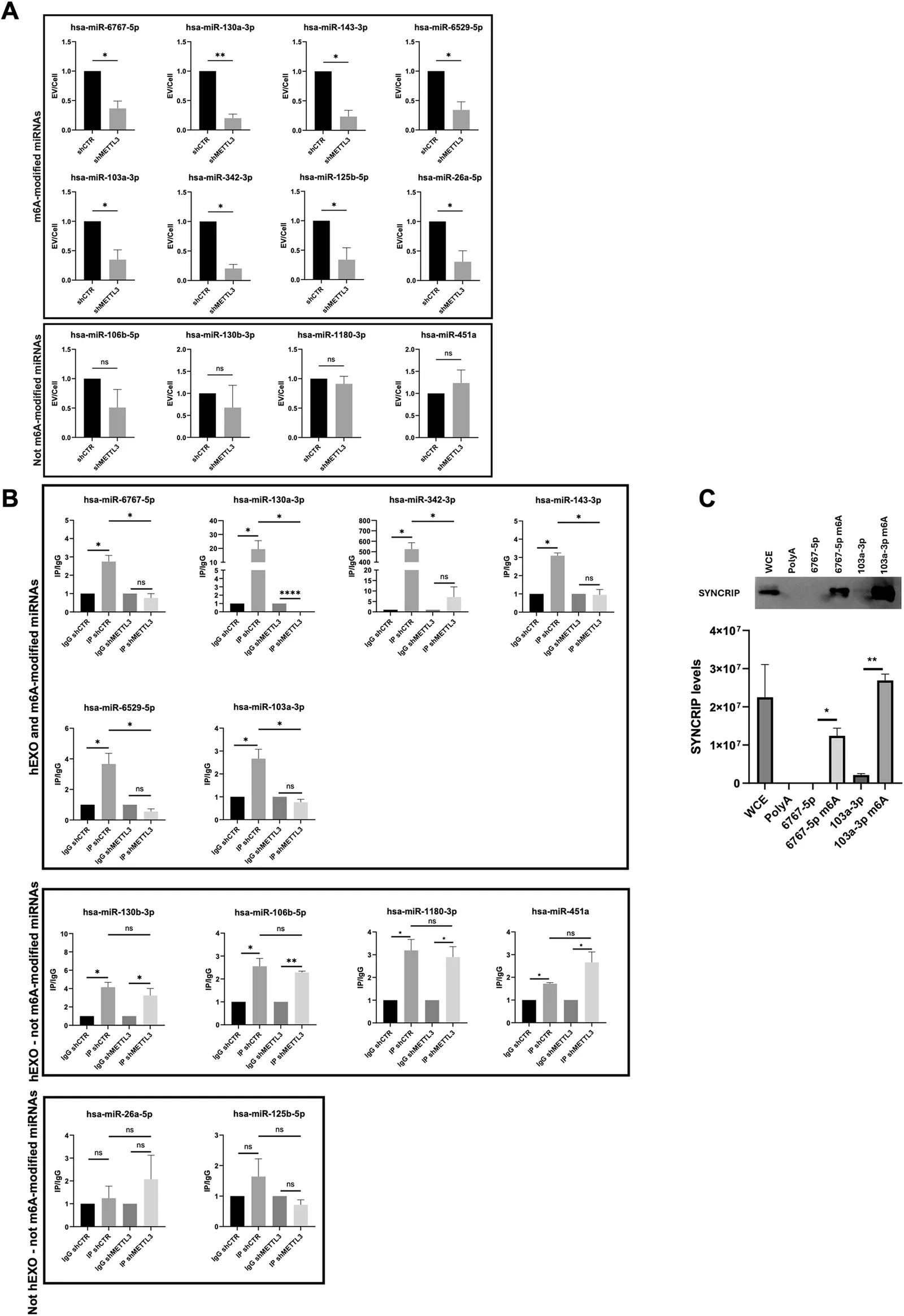
m6A facilitates miRNAs’ sEV-export and plays a role in promoting SYNCRIP binding. **A** miRNA levels in sEVs derived from shCTR and shMETTL3 HepG2 cells analyzed by RT-qPCR. To ensure that data regarding mature miRNAs methylation and sorting rather than a general defect in miRNA biogenesis or stability, data are expressed as a ratio of miRNA expression in sEVs with respect to the intracellular compartment (shCTR arbitrary value 1). **B** CLIP of SYNCRIP protein in HepG2 cells, both shCTR or shMETTL3. RT-qPCR analysis for the indicated miRNAs is shown as IP/IgG for each independent experiment (IgG arbitrary value 1). **C** (Top) Western blot assay on samples obtained by RNA pull-down with the methylated and unmethylated miR-6767-5p and miR103a-3p, followed by western blot for SYNCRIP. Data are representative of three independent experiments. PolyA has been used as a negative control. (Bottom) Quantification of SYNCRIP signal upon miRNA Pull-down followed by Western blot analysis. **A**–**C** Data are the mean ± S.E.M. of three independent experiments. Data are considered statistically significant with *p* < 0.05 (Student’s *t*-test). (*p < 0.05; **p < 0.01; ns not statistically significant).

To evaluate whether the methylation could have an impact on the same miRNAs’ export within the sEVs, as previously demonstrated [30], sEVs were isolated from control and METTL3-silenced HepG2 cells (Fig. 5A–D), and miRNAs’ export levels were assessed through RT-qPCR. Consistent with previous observation obtained upon SYNCRIP depletion, the abundance of the selected miRNAs was markedly reduced in sEVs from METTL3-silenced cells (Fig. 6A). To ensure that the data regard mature miRNA methylation and sorting rather than a general defect in miRNA biogenesis or stability, the ratio of mature miRNA levels in sEVs relative to their respective intracellular expression was calculated. This normalization specifically isolates the “loading efficiency” from the total cellular expression and is not impacted by potential variation in biogenesis and stability of specific miRNAs. Furthermore, this reduction was more evident than upon SYNCRIP silencing (Fig. 2F).

While both SYNCRIP and METTL3 were successfully depleted, we observed that METTL3 knockdown was more pronounced. This conceivably reflects the fact that while the enzyme METTL3 requires near-complete depletion to abrogate m6A catalytic activity, the RBP SYNCRIP may exert significant stoichiometric effects even with a partial reduction in protein levels. Despite the less pronounced knockdown of SYNCRIP, the resulting phenotypic changes (e.g., protein and RNA expression and migration ability) were statistically significant and followed the same trend as METTL3.

Thus, the hypothesis that METTL3-dependent m6A methylation might serve as a pivotal regulatory layer enabling the interaction between SYNCRIP and its target miRNAs and promoting their selective export into sEVs is conceivable.

To test this hypothesis, SYNCRIP CLIP was carried out in both control and METTL3-silenced cells. As expected, in control cells SYNCRIP efficiently binds to target miRNAs embedding the hEXO motif, whereas this interaction is strongly impaired in METTL3-silenced cells (Fig. 6B). As controls, miRNAs embedding the hEXO motifs but not m6A-modified and miRNAs methylated but not embedding the hEXO motifs were also analyzed (Fig. 6B).

Moreover, to determine whether SYNCRIP functions as an RBP that interacts with miRNA targets in a methylation-dependent manner or serves as a novel m6A “reader,” an RNA pull-down assay was performed. Specifically, synthetic biotinylated miRNA mimics were synthesized in both their unmethylated and m6A-methylated forms. Following incubation with protein extracts derived from HepG2 cells, Western blot analysis revealed that SYNCRIP is specifically bound to the methylated miRNAs. Conversely, the absence of m6A modification significantly impaired the interaction (Fig. 6C). These findings demonstrate that SYNCRIP selectively recognizes m6A-methylated miRNAs, defining its role as a reader in the context of HCC-derived small RNAs, ultimately driving their sorting into sEVs.

Several RBPs, including hnRNPA2B1 and PCBP2, have been characterized for their roles in the selective loading of miRNAs into sEVs or their regulated intracellular retention. Thus, it was investigated whether SYNCRIP might function in complex with these regulators. To test this hypothesis, co-immunoprecipitation (co-IP) assays were performed in HepG2 cells to evaluate the potential interaction between SYNCRIP and both hnRNPA2B1 and PCBP2. The results demonstrated an interaction between SYNCRIP and PCBP2, consistent with previous findings [26]. In contrast, no detectable interaction was observed between SYNCRIP and hnRNPA2B1 under the same experimental conditions (Supplementary Fig. 2A).

To address the mechanistical interplay between SYNCRIP and hnRNPA2B1, competitive/cooperative binding assays were performed. The CLIP results (Supplementary Fig. 2B) show that A2B1 is not significantly displaced/recruited by modulating SYNCRIP expression levels. This suggests that SYNCRIP and A2B1 recognize miRNAs (and specifically the m6A-site) through an autonomous mechanism. This is also in line with a previous report showing that although SYNCRIP and hnRNPA2B1 are members of a common EV-sorting complex, the two proteins display sequence-specific EV-sorting capacity in the loading of selected miRNAs [12]. Moreover, CLIP experiments were performed in HepG2 cells silenced for SYNCRIP to investigate the dynamics of PCBP2 binding. Notably, PCBP2 recruitments on miRNAs embedding both the CELL and the hEXO motifs require SYNCRIP binding in line with [26] (Supplementary Fig. 2C).

Overall, the depletion of one RBP does not necessarily abolish the binding of the other, pointing toward a model where these proteins may coordinate and recognize different structural or sequence-specific motifs on specific miRNA populations in a hierarchical or alternative manner.

### EMT is impacted by sEV methylated miRNAs

**Fig. 7:**
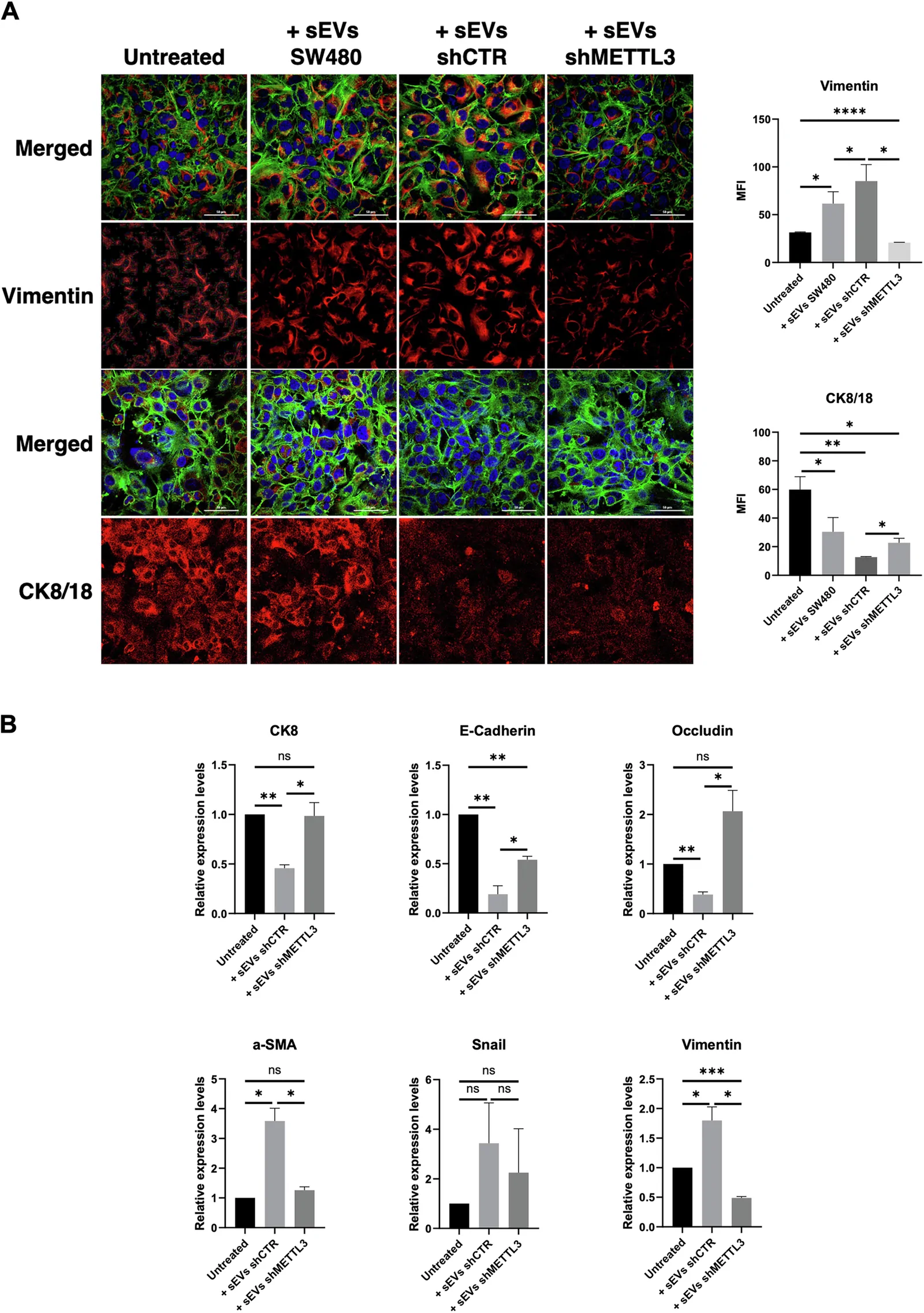
EMT is impacted by sEV methylated miRNAs. **A** (Left) Immunofluorescence assay on THLE-2 hepatocytes treated with the indicated sEVs and labeled as in Fig. 2A. Results are representative of four independent experiments. Scale bar: 50 μm. (Right) Quantitative analysis of immunofluorescence signals (as in Fig. 3A). **B** RT-qPCR for the indicated EMT markers on RNA samples from THLE-2 hepatocytes treated with the indicated sEVs. (Top) epithelial markers; (Bottom) mesenchymal markers. Data are shown as the mean ± S.E.M. of three independent experiments. **A**, **B** Data are considered statistically significant with *p* < 0.05 (Student’s *t*-test). (*p < 0.05; **p < 0.01; ***p < 0.001; ns not statistically significant).

To assess the impact of the depletion of the methylation machinery on sEV-mediated EMT in recipient cells, sEVs derived from METTL3-silenced HepG2 cells and relative controls were used to treat THLE-2 hepatocytes. Recipient cells treated with shCTR cell-derived sEVs undergo EMT, as evidenced by a marked downregulation of the epithelial marker CK8/18 and a concomitant upregulation of the mesenchymal marker vimentin (Fig. 7A). Conversely, hepatocytes treated with sEVs released from METTL3-depleted cells fail to undergo EMT. The analysis of recipient cells’ gene expression was also extended to further EMT-related markers by qRT-PCR analysis, highlighting the ability of shCTR HepG2-derived sEVs to downregulate the expression of epithelial markers (CK8, E-cadherin, and Occludin) and to induce the expression of mesenchymal markers (Vimentin, Snail, and α-SMA). More interestingly, data indicate that this ability is impaired in the case of treatment with sEVs produced by METTL3-silenced HCC cells (Fig. 7A, B).

These findings provide compelling evidence that METTL3-dependent m6A methylation plays a crucial role in determining the molecular profile of sEV-miRNA cargo, and consequently, the functional properties of the secreted sEVs.

## Discussion

sEVs condition tumor-TME communication through the selective and regulated miRNA delivery; this process, only partially disclosed, is controlled by RBPs and RNA modifications.

In this frame, the conceptual advances of this manuscript are: (i) SYNCRIP plays a key role in shaping the TME by modulating miRNA sEV informational cargo; (ii) this function is impacted also by the epitranscriptomic modification mediated by METTL3, providing an additional regulatory layer for SYNCRIP recruitment other than sequence specificity. Thus, these findings qualify SYNCRIP as a novel m6A reader. Moreover, (iii) epitranscriptomic modifications confer specificity of miRNA sEV-loading, enhancing the function of at least two RBPs: here SYNCRIP is shown to act similarly to the already reported A2B1 [30], and (iv) METTL3 silencing in HCC cells deeply unpairs sEV-dependent epithelial genes’ repression and mesenchymal genes’ induction.

Tumor development is driven by both intrinsic alterations of cancer cells as well as by a dynamic communication between the tumor mass and the surrounding niche, constituting the TME [42, 43]; in this frame, sEV miRNA-cargo emerged as a key component influencing gene expression in paracrine and endocrine intercellular signaling during cancer progression [44–46].

It has been extensively reported that sEV miRNA cargo is cell type-specific and varies depending on the pathophysiological status of the cell [14, 30, 47, 48], and several RBPs have been identified as crucial regulators of miRNA export or their intracellular retention [11, 12, 14, 26]. Alongside the protein-mediated mechanisms, it has also been highlighted that the existence of different sequence determinants in the miRNAs is required for miRNA-RBP interaction and functionally for miRNA export/retention [11, 12, 14, 26].

In addition to the RBPs/sequence motifs-based mechanism driving miRNA sEV-loading, a recent work disclosed the role of epitranscriptomic modifications [30]. Specifically, m6A modification has been identified in mature miRNAs where it acts as a negative regulator of their intracellular function while being instrumental to hnRNPA2B1-mediated miRNA export [30].

All this evidence supported the hypothesis of multiple selective mechanisms beyond the export of miRNAs into sEVs that are finely regulated and interconnected.

Here, the functional interplay between the RBP and miRNA EV-loader SYNCRIP and m6A has been dissected. Besides the previously mentioned role in the partition of miRNAs in non-tumorigenic hepatocytes [12], previous reports showed SYNCRIP’s role in HCC cells, facilitating EMT [22]. Thus, the role of SYNCRIP in determining the informational cargo of sEVs in HCC cells, the SYNCRIP-dependent sEV-mediated EMT induction, and the interplay between miRNA methylation and SYNCRIP binding were assessed. This work identifies a novel epitranscriptomic checkpoint for intercellular communication, demonstrating for the first time that the METTL3-dependent m6A modification acts as a “molecular barcode” that SYNCRIP recognizes to facilitate the selective packaging of miRNAs into sEVs.

First, SYNCRIP-dependent miRNA sEV-loading was characterized by NGS analysis, and a motif search on miRNAs highlighted the presence of a full hEXO motif, in line with previous evidence [12].

Furthermore, since a GO term and a KEGG analysis on the putative targets of these miRNAs highlighted an enrichment of terms related to the EMT process, the SYNCRIP sEV-mediated pro-EMT activity was addressed. Notably, non-tumorigenic hepatocytes undergo EMT upon the treatment with shCTR HCC-derived sEVs; conversely, this effect is limited upon the treatment with shSYNCRIP HCC-derived sEVs.

Moreover, since several SYNCRIP-target miRNAs undergo m6A modification [30], the impact of m6A on sEV-miRNA cargo was assessed: coherently to previous findings, the sEV-loading of these miRNAs is deeply impaired by METTL3 depletion.

In addition, the analysis of miRNA-target mRNAs highlighted an enrichment of EMT-related terms, and the evaluation of the impact of sEVs produced by HCC cells on non-tumorigenic hepatocytes indicated a significant induction of the EMT program, which requires METTL3.

The overlap among SYNCRIP-dependent sEV-loaded and methylated miRNAs led to investigating the direct role of SYNCRIP as an m6A reader. Notably, m6A modification is required for SYNCRIP binding to several miRNAs, indicating that the presence of sequence motifs is not the unique determinant allowing miRNA-RBP interaction and that RNA methylation is a prerequisite for the recruitment of distinct RBPs (i.e., SYNCRIP and A2B1).

In this scenario, the identification of the precise domain of SYNCRIP responsible for m6A recognition is also a significant issue; determining which specific domain (or combination of domains) is responsible for this m6A-dependent affinity, while it would require extensive structural studies, will shed light on the structural dynamics of interaction. While our evidence suggests that SYNCRIP might interact with m6A-modified miRNAs directly, it also appears conceivable that the interaction occurs through a multi-protein complex involving other canonical readers (e.g., YTHDF family), providing a more complex mechanistic model.

Co-IP experiments aiming at clarifying the biochemical interplay within the sorting complex confirm SYNCRIP-PCBP2 binding, while no direct interaction was detected between SYNCRIP and hnRNPA2B1. Mechanistically, SYNCRIP silencing impairs PCBP2 recruitment on miRNAs containing both CELL and hEXO motifs. This suggests a hierarchical model where SYNCRIP acts as a primary driver for PCBP2 recruitment (Supplementary Fig. 2C). In contrast, competitive binding assays revealed that hnRNPA2B1 recruitment is independent of SYNCRIP levels (Supplementary Fig. 2B), indicating that these two RBPs recognize miRNAs (specifically m6A sites) through autonomous mechanisms.

These data align with evidence that SYNCRIP and hnRNPA2B1, despite belonging to a common sEV-sorting complex, maintain distinct sequence-specificities [12]. Overall, these findings demonstrate that these RBPs recognize different structural or sequence-specific motifs independently, establishing SYNCRIP as a primary and sufficient driver of the sorting process described here.

These findings may pave the way for the development of therapeutic strategies counteracting the activity of this RBP, aiming at the control of sEV informational content in terms of miRNAs and therefore of their function. However, a limitation of this approach is that these RBPs are involved in multiple essential cellular processes, such that their depletion may lead to off-target effects in sEV-producing cells.

Since RNA methylation acts upstream of RBP/miRNAs interaction, determining miRNA sEV-loading, the impairment of METTL3 activity may represent a more advantageous strategy.

While our in vitro findings suggest that the METTL3-SYNCRIP axis plays a critical role in HCC progression, further investigation using in vivo models and pharmacological inhibitors is necessary to determine the safety and efficacy of targeting these candidate proteins for future translational research from the perspective of a therapeutic setting.

Overall, the clarification of these dynamics is crucial in terms of knowledge improvement as well as in terms of the design of sEVs with an ad hoc informational content from the perspective of sEV-based therapeutic strategies.

## Supplementary information

Supplementary Material

Supplementary material: ROw NGS data

Original Data

## Data Availability

NGS data have been deposited in NCBI’s Gene Expression Omnibus and are accessible through GEO Series accession number GSE317620 (https://www.ncbi.nlm.nih.gov/geo/query/acc.cgi?acc=GSE317620). NCBI Sequence Read Archive (SRA) accession has been released and will be available as soon as possible.
