# Supplementary material for: Interplay between the RBP SYNCRIP and RNA methylation in determining sEV miRNA-cargo and function in cell-to-cell communication
Source: Cell Death Dis. 2026 Jun 15;17(1):749. doi: 10.1038/s41419-026-08991-9 (PMC13500694; doi:10.1038/s41419-026-08991-9)
Supplement: Supplementary file 1 [file 41419_2026_8991_MOESM1_ESM.pdf]

SUPPLEMENTARY INFORMATION

SUPPLEMENTARY MATERIALS AND METHODS

Co-immunoprecipitation

Cells were lysed with IP Lysis Buffer (150 mM NaCl, 50 mM Tris-HCl pH 7.5, 5 mM EGTA pH 8, 50 mM NaF pH 8, 1,5 mM MgCl2, 1% TRITON-X100, and 10% glycerol) containing freshly added cocktail protease inhibitors (complete EDTA-free Protease Inhibitor Cocktail; Sigma-Aldrich) and phosphatase inhibitors (5 mM EGTA pH 8.0; 50 mM sodium fluoride; 5 mM sodium orthovanadate). Lysates were incubated on a rotating platform for 2 h at 4°C and then centrifuged at 13,000 rpm for 30 min at 4°C. Protein concentration was determined with Protein Assay Dye Reagent (Bio-Rad), based on the Bradford assay. 2 mg of proteins (one for the specific antibody and one for the corresponding aspecific IgG) were precleared adding 40 µL of Protein A Sepharose or Protein G Sepharose (GE HealthCare) for 3 hr at 4°C in a total volume of 1 mL of IP Lysis Buffer in rotation. Then, Protein G Sepharose was removed by centrifugation and the extracts were incubated with 5 µg of specific antibody α-SYNCRIP (MAB11004; Merck Millipore), or Normal Mouse IgG (12-371; Merck Millipore), used as negative control, to proceed with immunoprecipitation at 4°C overnight. Immuno-complexes were collected adding 50 µL of Protein G Sepharose for 3 h at 4°C in rotation. The immunoprecipitated proteins were washed three times with Net Gel Buffer (150 mM NaCl, 50 mM Tris-HCl pH 7.5, 1 mM EDTA, 0.1% NP40, and 0.25% gelatin) and once with RIPA Buffer (150 mM NaCl, 1% NP40, 0,5% sodium deoxycholate, 0,1% SDS, 50 mM Tris-HCl pH 8). Finally, immunoprecipitated proteins were detached from Sepharose beads by adding 50 µL of Laemmli Buffer 2X. Samples were boiled at 95°C for 5 min, beads were eliminated by centrifugation, and 10% of input sample and 50% of each immunoprecipitated sample were loaded on polyacrylamide gel and analyzed by western blotting.

miRNA mimics' transfection

For miRNA mimics ectopic expression, THLE-2 cells were transfected with GenePharma® miRNA mimics -hsa-miR-130b-3p (sequence 5'CAGUGCAAUGAUGAAAGGGCAU3')-hsa-miR-106b-5p (sequence 5'UAAAGUGCUGACAGUGCAGAU3')-hsa-miR-103a-3p
(sequence 5'AGCAGCAUUGUACAGGGCUAUGA3') and the same amount of miRNA mimic Negative Control. RNAiMAX Reagent from Thermo Fisher Scientific (Waltham, MA USA) was used as transfection agent.

Analyses have been performed 24h after transfection. miRNAs were extracted by miRNeasy Mini Kit and RNeasy MinElute Cleanup Kit (QIAGEN, Germany), and reverse transcribed with microScript microRNA cDNA Synthesis Kit (Norgen Biotek Canada). Quantitative polymerase chain reaction (RT-qPCR) analyses were performed according to MIQE guidelines (40,41). cDNAs were amplified by RT-qPCR reaction using GoTaq qPCR Master Mix (Promega Corporation, USA). Relative amounts, obtained with the 2^(-ΔCt) method, were normalized with respect to U6 snRNA.

Total RNA was extracted by ReliaPrepTM RNA Tissue Miniprep System (Promega Corporation, USA) and reverse transcribed with iScriptTM c-DNA Synthesis Kit (Bio-Rad Laboratories Inc., USA). cDNAs were amplified by RT-qPCR reaction using GoTaq qPCR Master Mix (Promega Corporation, USA). Relative amounts, obtained with the 2^(-ΔCt) method, were normalized with respect to the housekeeping gene L32.

SUPPLEMENTARY FIGURE LEGENDS

Supplementary Figure 1: miRNA mimics corresponding to different miRNAs loaded in HepG2-derived sEVs negatively regulate EMT-related target genes.

qRT-PCR analysis of miRNA mimics expression after transfection (*top*) and of intracellular mRNA levels of the indicated markers (*bottom*) after transfection of THLE-2 with the indicated miRNA mimics. mRNA values are calculated by the ΔΔCt method, normalized to the RPL32 ribosomal RNA levels and shown as means ± SEM. The levels of mimic expression were normalized to the U6 levels

and shown as means ± SEM. Statistical analysis was performed by Student's T-test (N=3). $P \leq 0.05$ is considered statistically significant. (*$p \leq 0.05$; **$p<0.01$; *** $p<0.001$).

**MicroRNAs**	**miRBase ID**	**Target genes validated upon mimics' transfection**
hsa-103a-3p	MIMAT0000101	E-cadherin, CK18, Occludin
hsa-miR-106b-5p	MIMAT0000680	E-cadherin, CK8, Occludin
hsa-miR-130b-3p	MIMAT0000691	E-cadherin, Occludin

Supplementary Table 1: List of miRNAs analyzed with their target genes (listed in the last column).

Supplementary Figure 2: SYNCRIP expression affects PCBP2 binding while A2B1-miRNA interaction is independent of SYNCRIP expression

A) Co-immunoprecipitation of SYNCRIP, PCBP2 and A2B1 in HepG2 cells. Data are representative of three independent experiments. WCE: whole cell extract. IgG: negative control of immunoprecipitation. IP SYN: sample immunoprecipitated with the antibody specifically recognizing SYNCRIP.

B) CLIP of A2B1 protein in HepG2 cells both shCTR (shCTR) or shSYNCRIP (shSYN). RT-qPCR analysis for the indicated miRNAs is shown as IP/IgG for each independent experiment (IgG arbitrary value 1). Data are shown as the mean ± S.E.M. of four independent experiments.

C) CLIP of PCBP2 protein in HepG2 cells both shCTR (shCTR) or shSYNCRIP (shSYN). RT-qPCR analysis for the indicated miRNAs is shown as IP/IgG for each independent experiment (IgG arbitrary value 1). Data are shown as the mean ± S.E.M. of four independent experiments.

B-C) Data are considered statistically significant with $p<0.05$ (Student's T test). (*: $p<0.05$; **: $p<0.01$; ***: $p<0,001$; ns: not statistically significant).

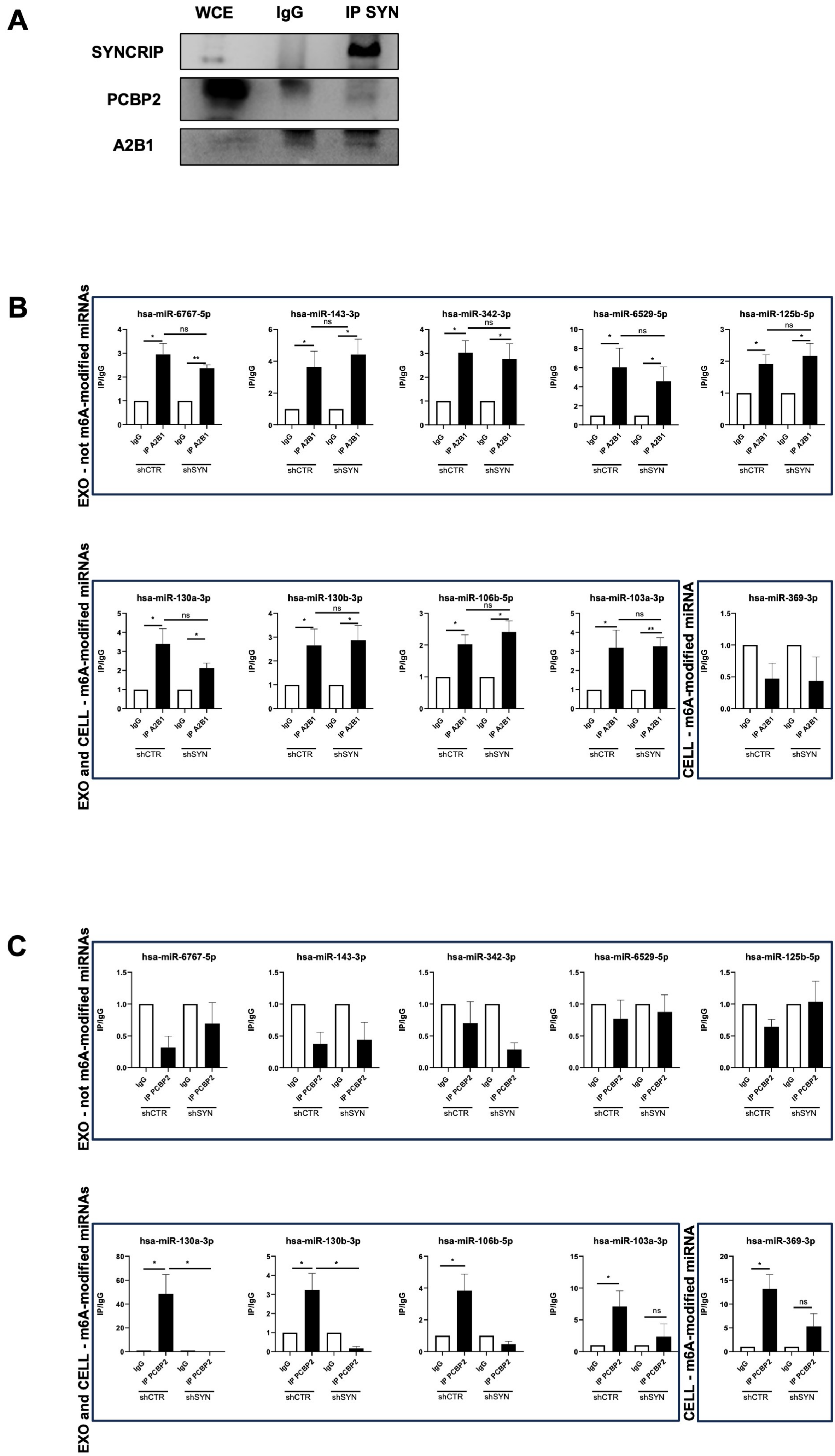

Supplementary Figure 1

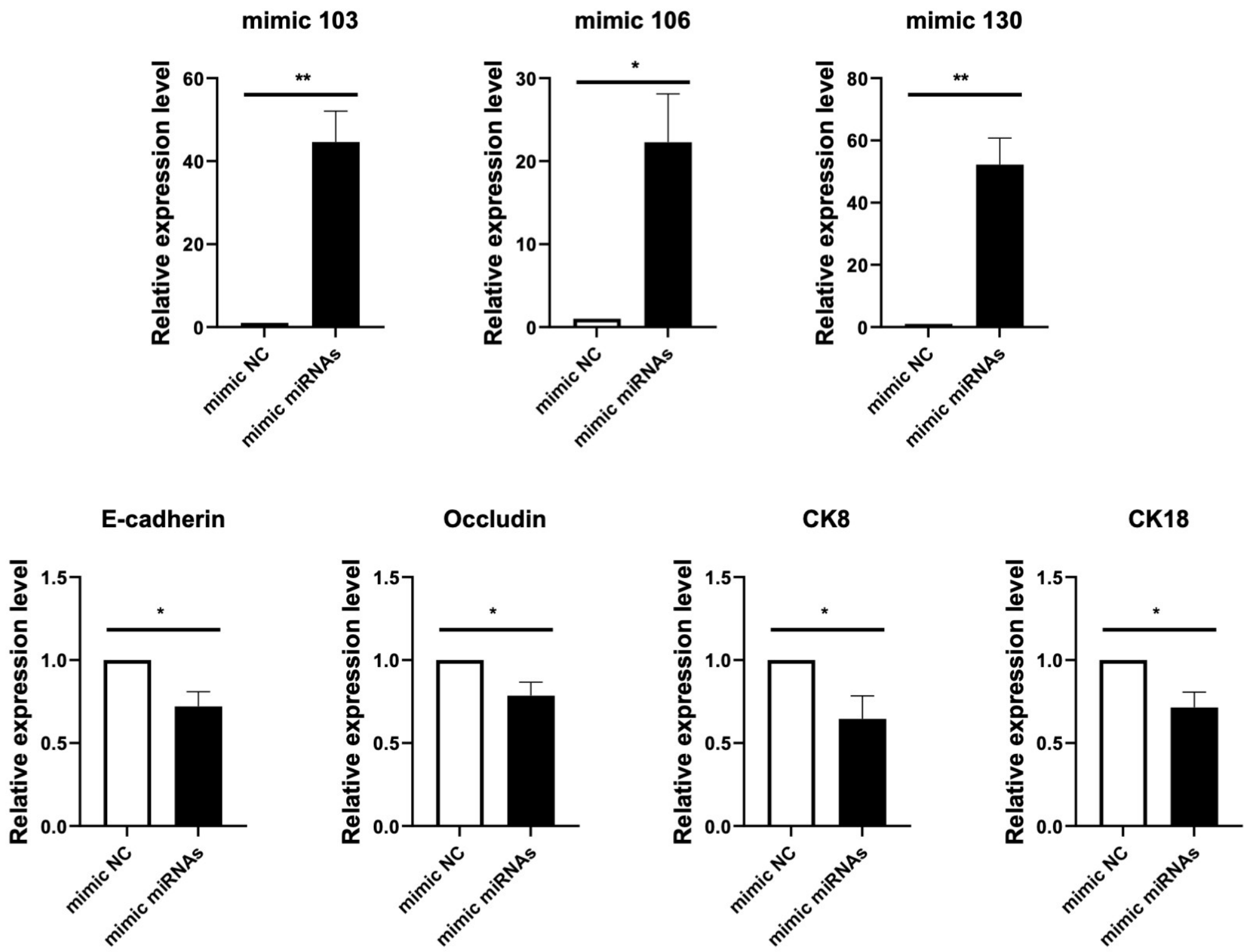

Supplementary Figure 2
