# Supplementary material for: Interplay between the RBP SYNCRIP and RNA methylation in determining sEV miRNA-cargo and function in cell-to-cell communication
Source: Cell Death Dis. 2026 Jun 15;17(1):749. doi: 10.1038/s41419-026-08991-9 (PMC13500694; doi:10.1038/s41419-026-08991-9)
Supplement: Supplementary file 2 [file 41419_2026_8991_MOESM3_ESM.pdf]

Western blots

Figure 1 panel B

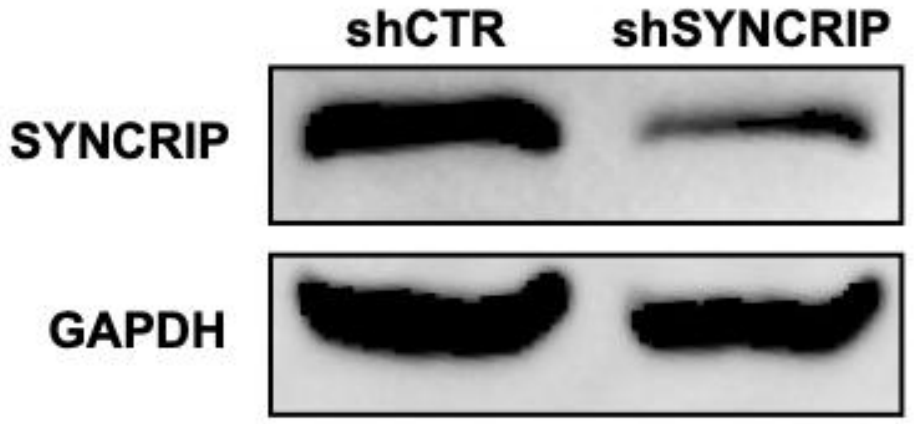

Original acquisition

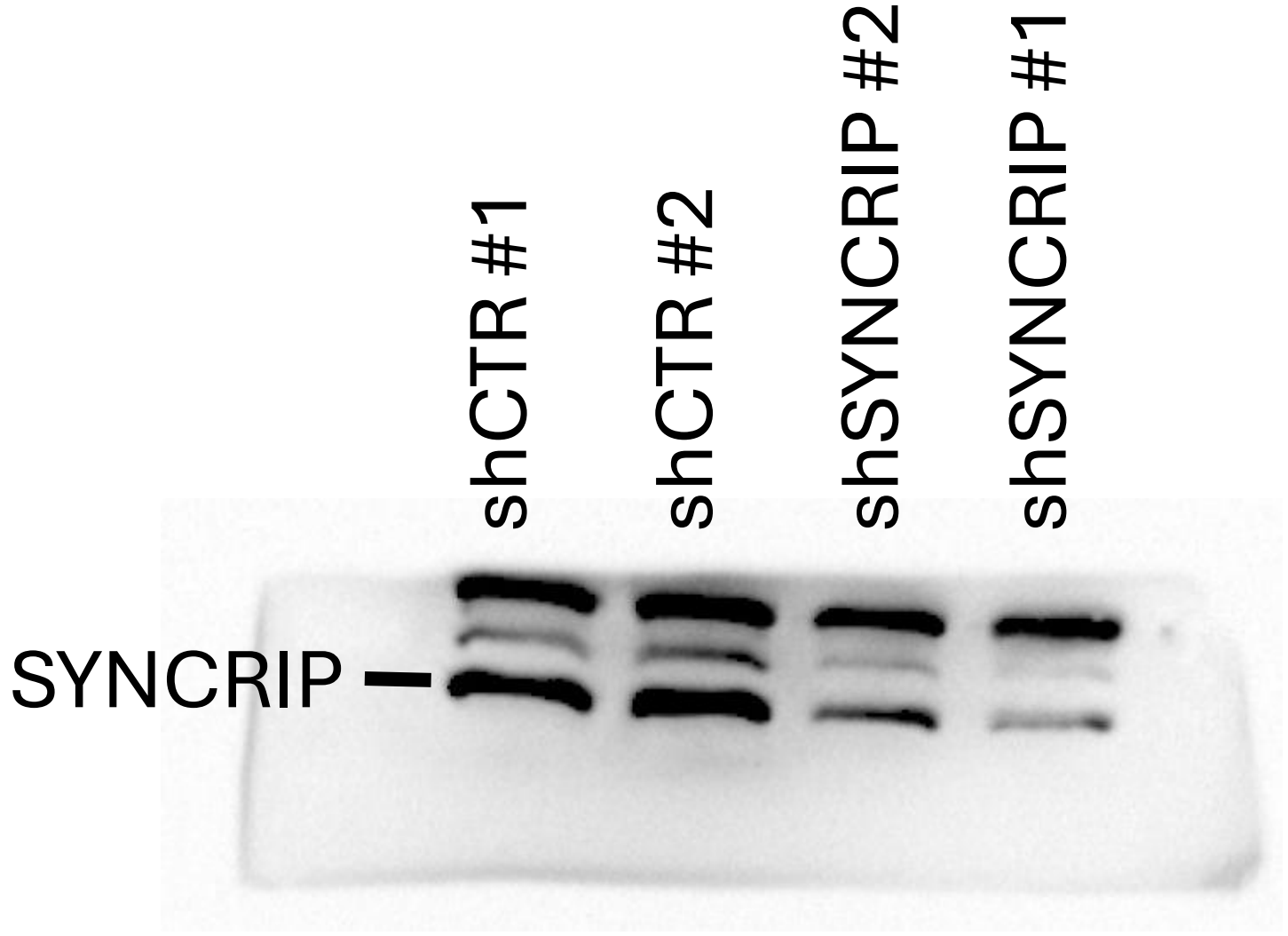

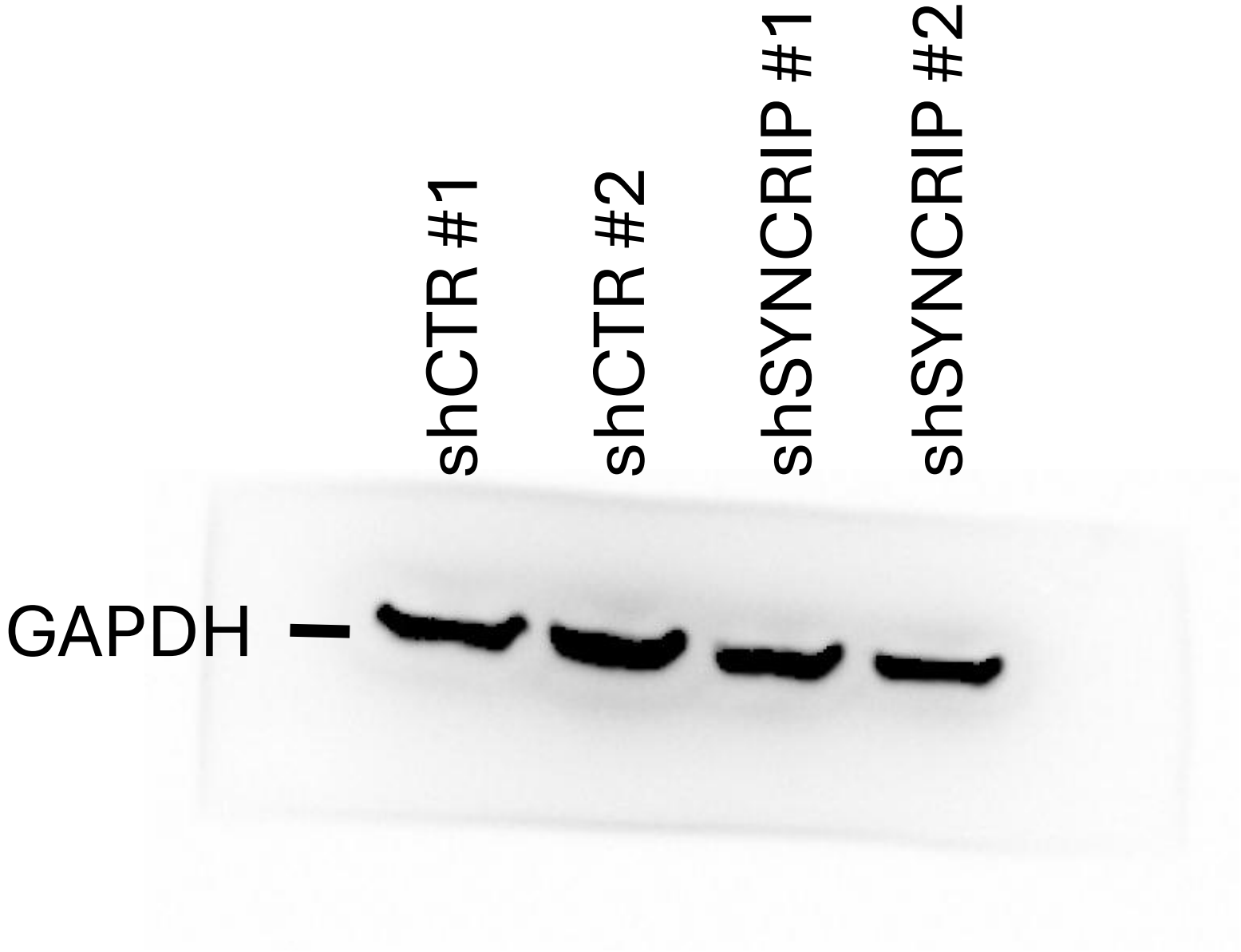

Western blots

Figure 5 panel B

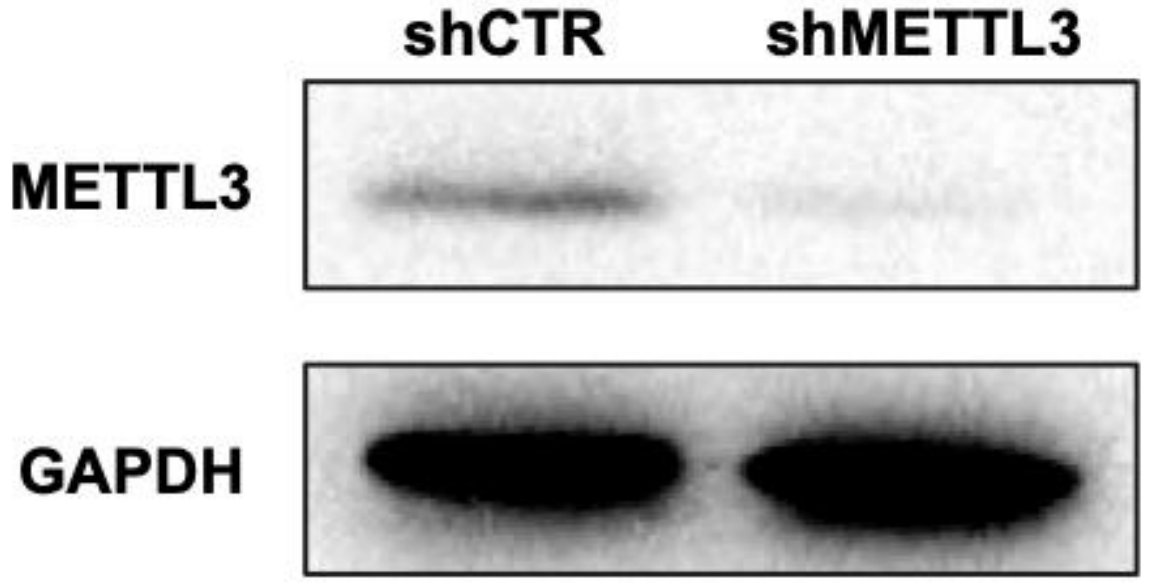

Original acquisition

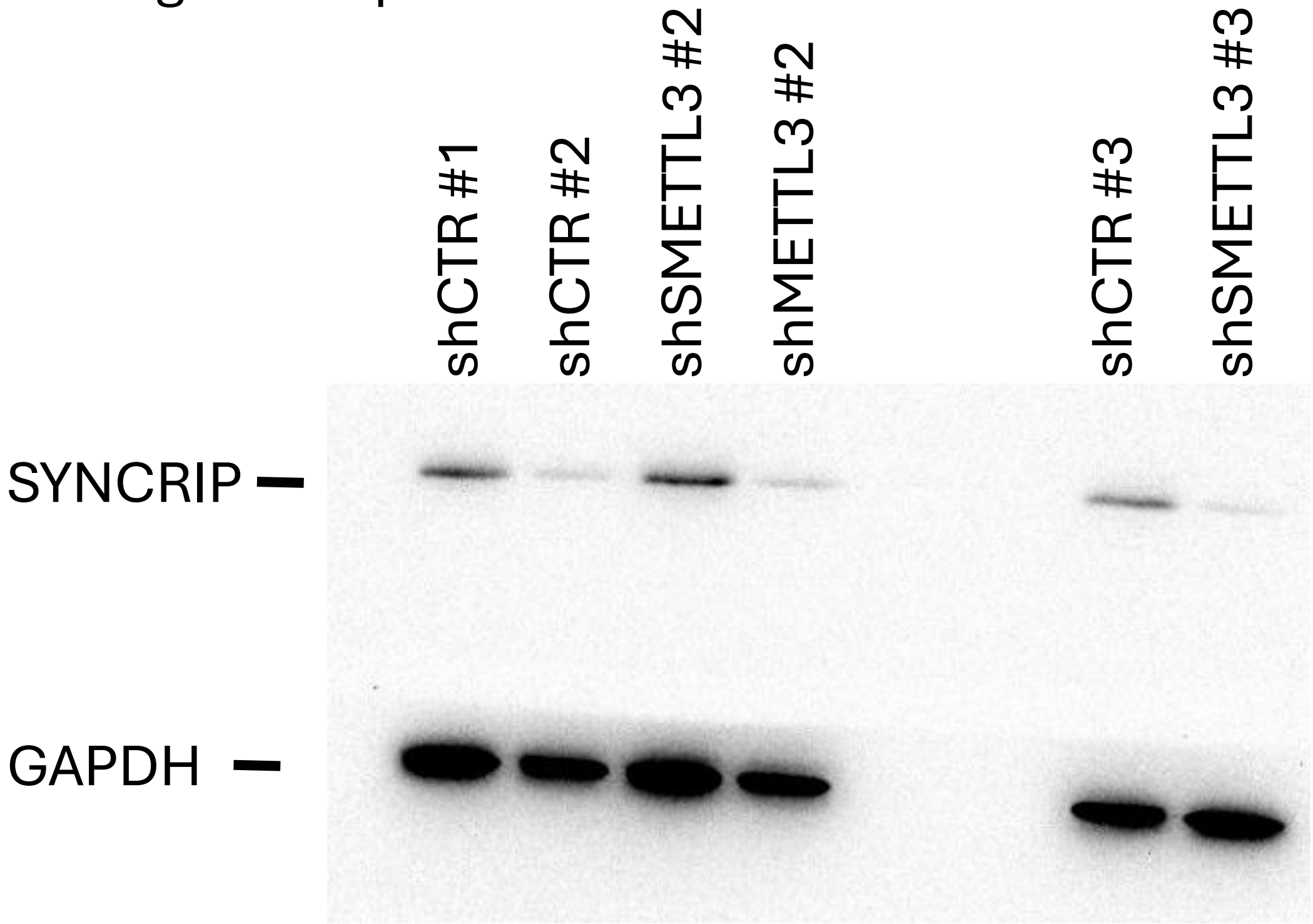

Pull down assays

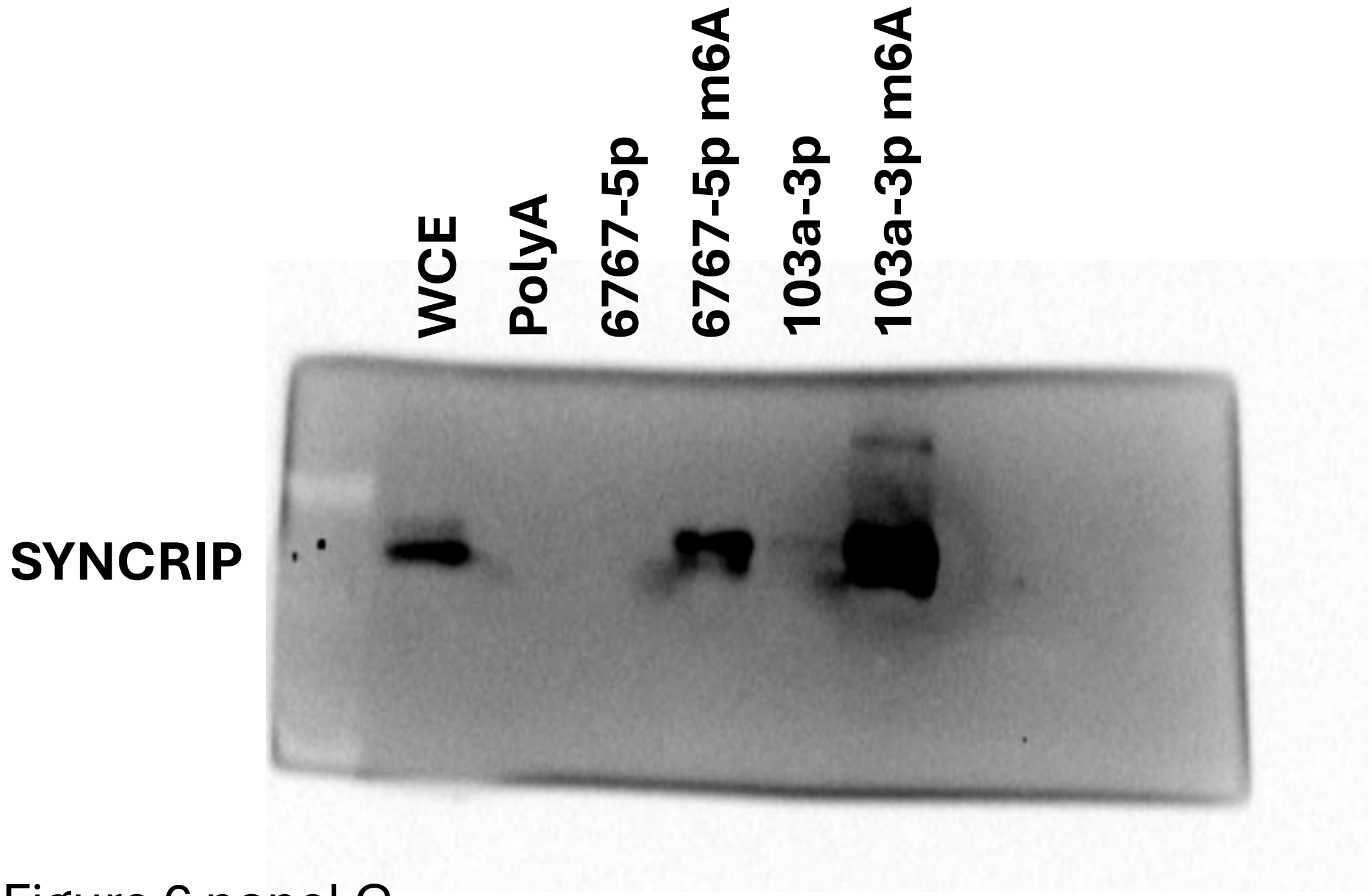

Figure 6 panel C

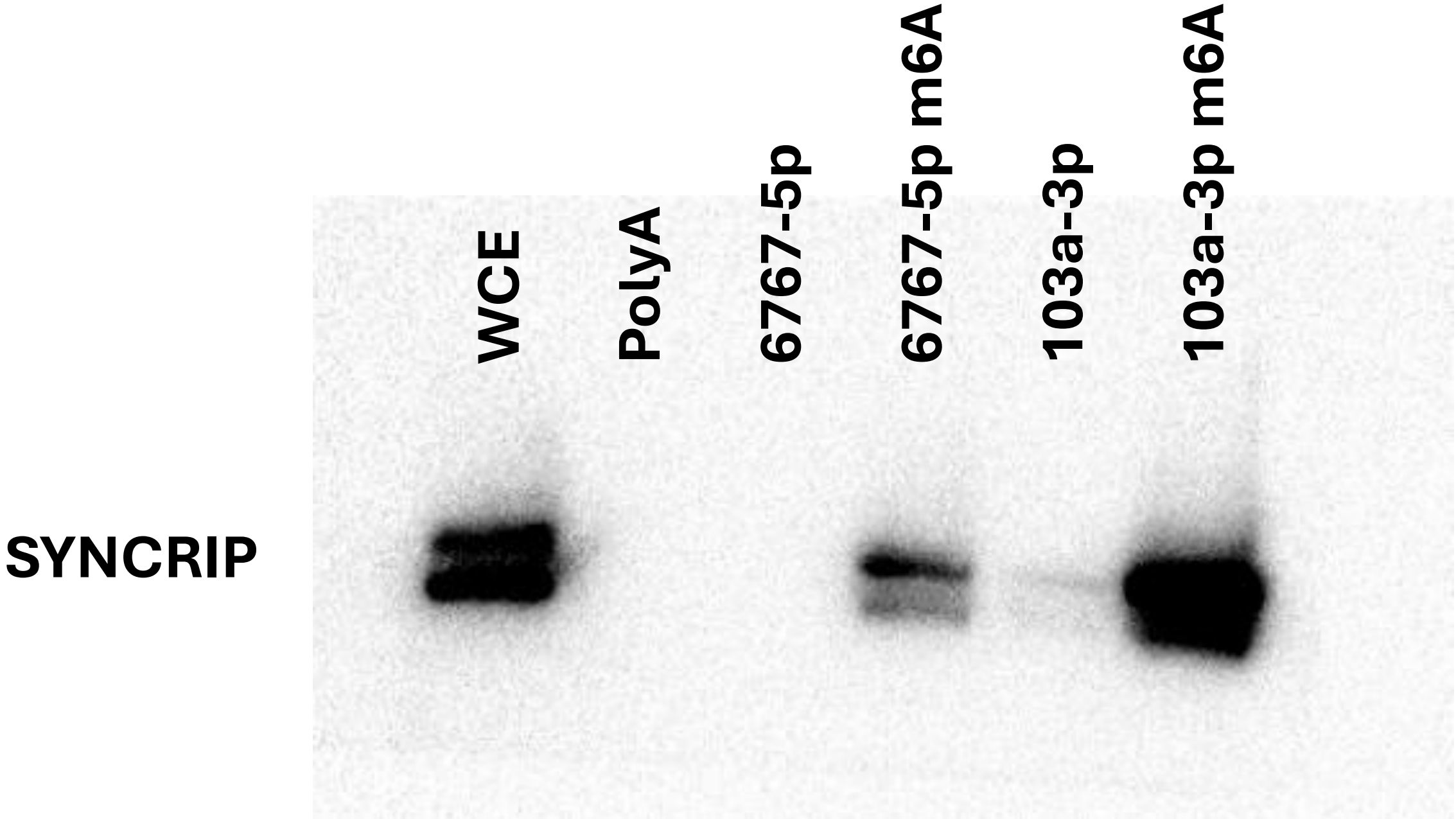

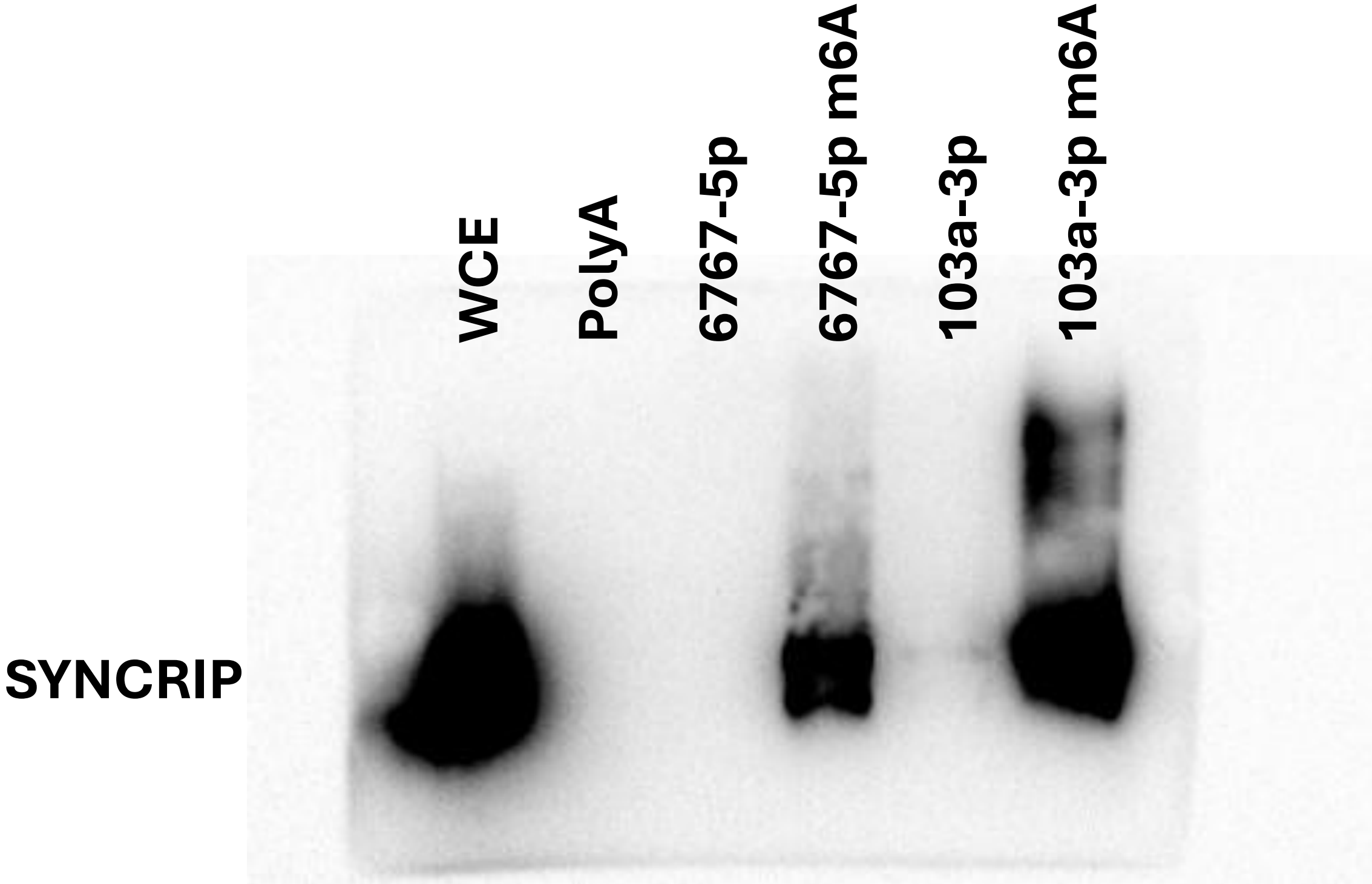

Co-IP assays

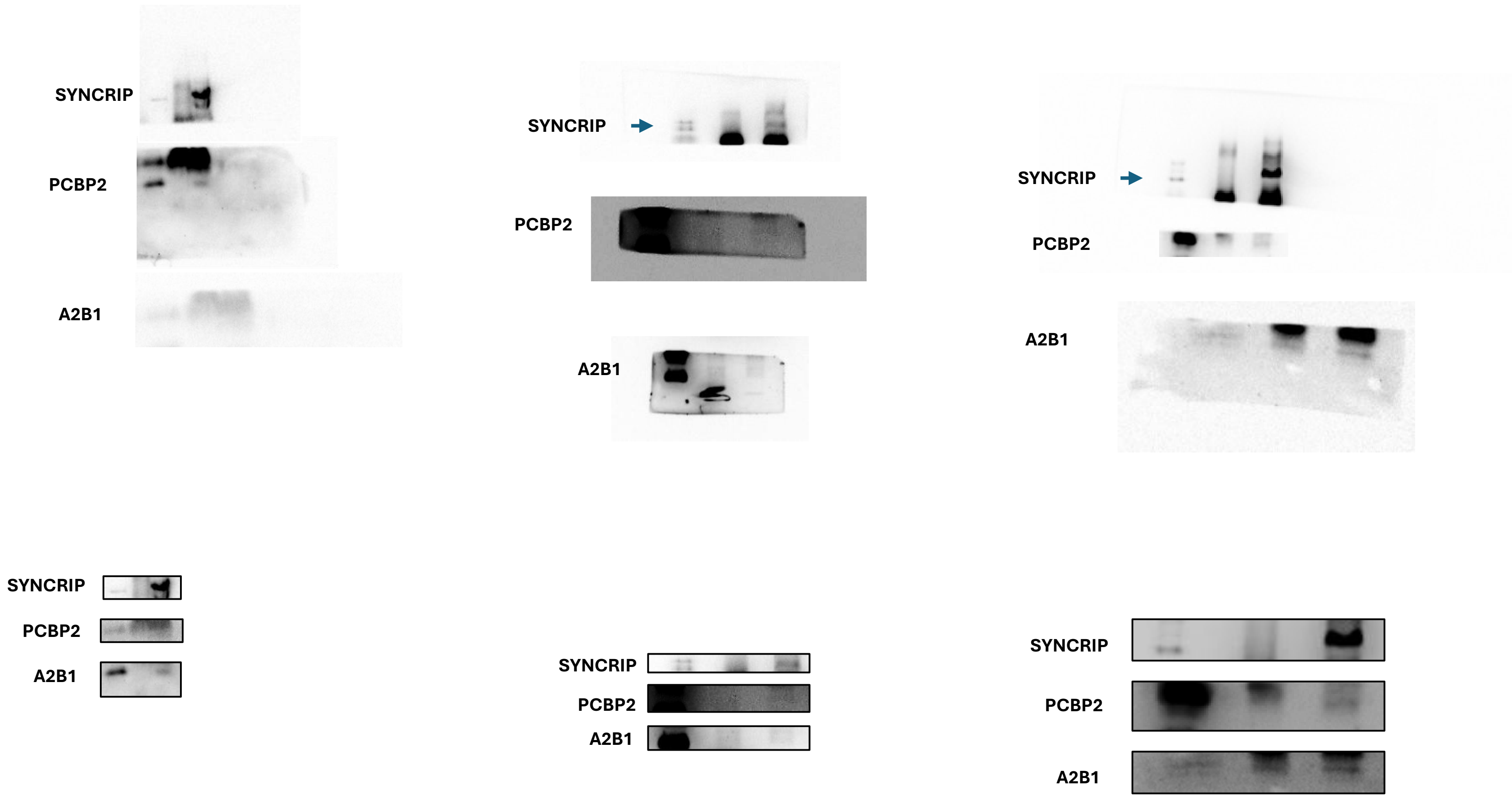

Figure S2 panel A

sEV markers

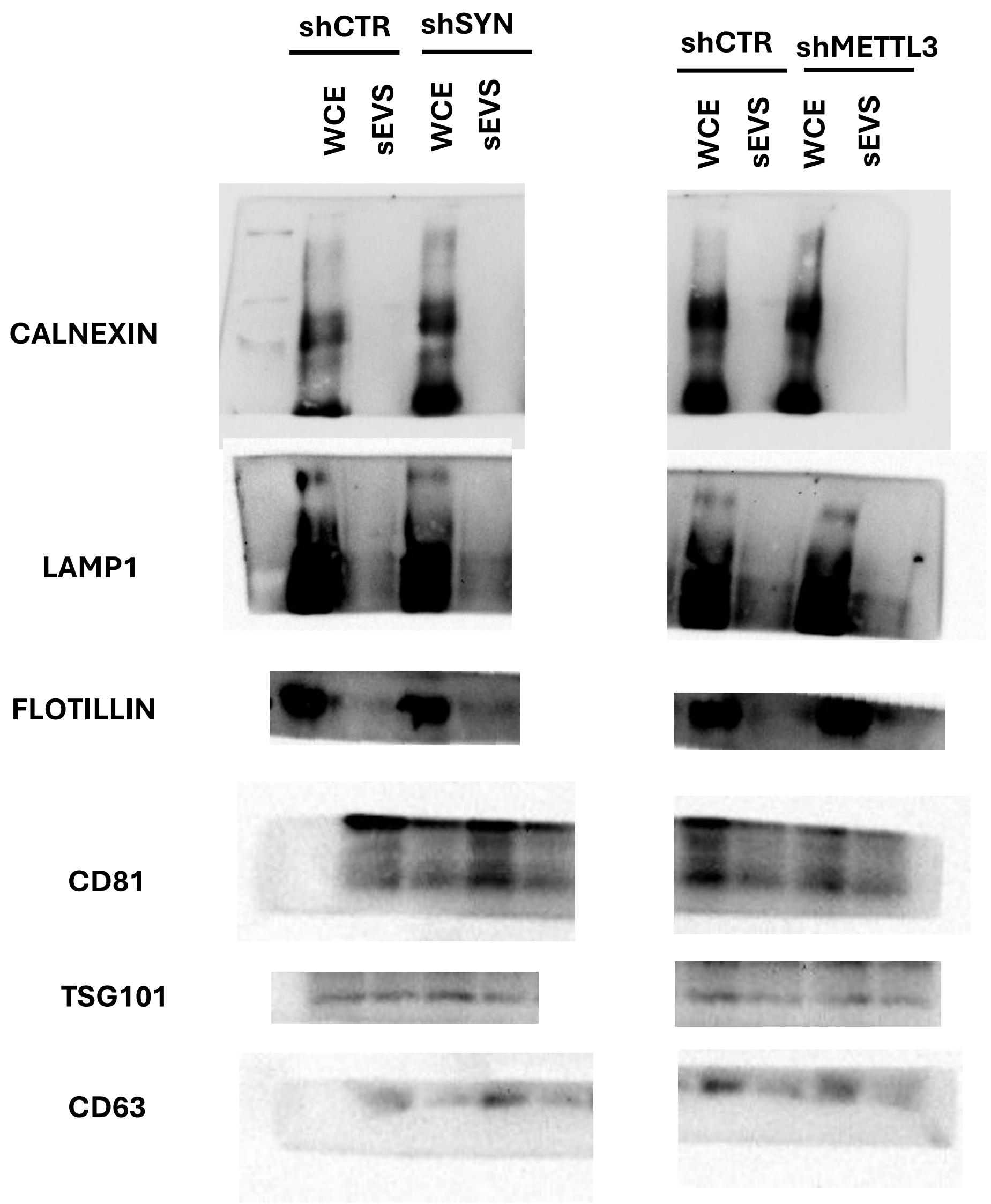

Figure 1 and 5 panels D
